# *FTX271*: A potential gene resource for plant antiviral transgenic breeding

**DOI:** 10.3389/fmicb.2022.1003478

**Published:** 2022-09-29

**Authors:** Yuhan Zhang, Chaoming Gao, Yahong Zhang, Hang Huang, Yameng Du, Lan Wu, Liping Wu

**Affiliations:** School of Life Science, Key Laboratory of Poyang Lake Environment and Resource, Ministry of Education, Nanchang University, Nanchang, Jiangxi, China

**Keywords:** tobacco mosaic virus, transgenic tobacco, disease resistance mechanism, omics, resistance gene

## Abstract

Flammutoxin (FTX), as well as its precursor TDP, is a protein from *Flammulina velutipes* with antiviral activity. Transgenic tobacco with the *FTX271* (gene of FTX or TDP) can not only delay the onset time of symptoms but also alleviate the symptoms caused by tobacco mosaic virus (TMV), but the mechanism is still unclear. In this study, *FTX271* was introduced into *Nicotiana benthamiana,* and the disease resistance mechanism activated by *FTX271* was speculated by transcriptomic and proteomic techniques. The results showed that TDP was detected, and some genes, proteins and pathways were significant upregulated or enriched in transgenic tobacco, including the mitogen-activated protein kinase (MAPK) cascade signal transduction pathway, the expression of hypersensitive response (HR) marker genes *H1N1* and *HSR203J*, pathogenesis-related (PR) genes, and the key genes *COI1* and lipoxygenase gene *LOX2* of the jasmonic acid (JA) signaling pathway, indicating *FTX271* may activate the MAPK pathway and increase the content of reactive oxygen species (ROS) and JA, which promoted the HR and inducible systemic resistance (ISR). ISR caused increased expression of peroxidase (POD) and other proteins involved in pathogen defense. In addition, transgenic tobacco may use sHSP-assisted photoreparation to alleviate the symptoms of TMV. In conclusion, JA-mediated ISR and sHSP-assisted photoreparation are activated by *FTX271* to protect tobacco from TMV infection and alleviate the symptoms caused by the virus. The study provided a theoretical basis for the TMV resistance mechanism of *FTX271*, which may represent a potential gene resource for plant antiviral transgenic breeding.

## Introduction

Tobacco (*Nicotiana tabacum* L.) is one of the most widely cultivated cash crops in China as well as worldwide (Zou et al., 2021). Many products, such as nicotine, solanesol, fiberboard, pulp, and organic fertilizers, can be generated from tobacco plants or their byproducts produced after processing (Hu et al., 2015; Banožić et al., 2020). Tobacco mosaic virus (TMV) is a positive-sense single-stranded RNA (ssRNA) virus of the genus Tobamovirus (Kobayashi et al., 2010). TMV-infected plants will exhibit various symptoms, ranging from shrunken, deformed and twisted leaves in mild cases to dwarfed plants in severe cases and abnormal flowering and fruiting, which seriously endangers the yield and quality of crops (Scholthof, 2004; Scholthof et al., 2011). Tobacco disease caused by TMV has brought much more losses to the tobacco economy than fungal and bacterial diseases, and it has become the biggest disaster that threatens tobacco production (Yuan et al., 2022).

Several strategies have been developed to control viral diseases, including transgenic technology. The gene transferred into plants can give the plants good antiviral properties, and the gene source can be the virus itself or the exogenous antiviral active protein (Suzuki et al., 1996; Baranwal and Verma, 1997). In 1986, Beachy introduced the coat protein (CP) gene of TMV into tobacco and obtained the first transgenic plant resistant to TMV infection (Reinero and Beachy, 1986). After that, other component genes of different viruses, such as replicase, protease, and mobile proteins, were also used as antiviral gene resources to control viruses. Nonviral-derived gene-mediated resistance has no risk of recombination and has higher safety. Therefore, nonviral transgenic technology is one of the research hotspots at this stage. At present, the main sources of nonviral genes are plant disease resistance-related genes, potential suicide genes, ribosomal inactivation protein genes, and other genes that can induce plant resistance (Lodge et al., 1993; Zheng et al., 2011).

Flammutoxin (FTX), a protein with a 251 amino acid (aa) sequence, is specifically expressed during the formation and growth of *Flammulina velutipes* fruiting bodies and has cardiotoxicity and cytolytic properties (Tadjibaeva et al., 2000; Jin et al., 2007). Transepithelial electrical resistance-decreasing protein (TDP) is the precursor of FTX, containing 271 aa, without cytotoxicity, but can promote paracellular permeability (Tomita et al., 2004). After analyzing the protein and nucleic acid sequences of TDP and FTX, Watanabe and Narai suggested that FTX is produced by cleaving the 20aa at the C-terminus of TDP during protein processing (Watanabe et al., 1999). Protein Zb extracted from *F. velutipes* can reduce the number of dead spots formed by TMV on *Nicotiana glutinosa* leaves (Fu et al., 2003). The aa sequence showed that the anti-TMV protein Zb may be FTX or TDP because its N-end is completely consistent with that of FTX and TDP. However, the function of FTX or TDP was only found when it acted on the cell membrane of animal cells or tobacco. To date, their real function during the formation of *F. velutipes* is still unclear.

Our previous study found that the onset time of symptoms caused by TMV was delayed and that mosaic symptoms were significantly reduced in *FTX271* transgenic tobacco (*N. tabacum* cv. K326), as well as in tobacco sprayed with the recombinant protein TDP (Wu et al., 2017; Han et al., 2022). Salicylic acid (SA)-mediated systemic acquired resistance (SAR) was induced by TDP to protect tobacco from TMV infection, but what causes mosaic symptoms to delay and lessen in *FTX271* transgenic tobacco? Is the transgene expression product TDP or FTX? JA and SA, triggered by the interaction of elicitor and plant receptor, are widely studied in plants, and both play an important role in plant defense signal transduction (Peng et al., 2005). The Nep1-like (NLPS) toxin family is a protein elicitor and generally induces immune responses and cell death in dicotyledonous plants. It is believed that NLPS can act as a cytolytic toxin to induce leakage of the cytoplasmic membrane, thus causing cytotoxicity (Santhanam et al., 2013). Is it possible that FTX also exploits its cytolytic toxin properties to induce plant immune defenses? FTX can promote the growth of fruiting bodies, does it have a plant growth-promoting effect?

To functionally characterize the *FTX271* gene during the transgenic tobacco response to TMV infection, transcriptomic and proteomic techniques were used to explore the effect on the expression of endogenous proteins in transgenic tobacco before and after infection with TMV. Additionally, p35s-30B-GFP was utilized to establish the transient expression system of TMV, and GFP was used as a reporter gene to indicate the expression of the virus in plants. The disease resistance of transgenic tobacco was further explored by determining the changes in the activity of defense enzymes. Based on the results of the joint action of multiple defense-related enzymes and metabolic pathways, we suggest that *FTX271* promotes the immunity of transgenic tobacco.

## Materials and methods

### Biomaterials and growth conditions

The seeds of *N. benthamiana* were preserved by our laboratory. *Escherichia coli* DH5α and Agrobacterium GV3101 were provided by our laboratory. The plant expression vector pROK II was purchased from Wuhan Miaoling Biological Company (Wuhan, China). The transient expression vector TMV-based p35s-30B-GFP was donated by Fujian Agriculture and Forestry University. Fresh fruiting bodies of *F. velutipes* were purchased from Nanchang, Jiangxi Province, China.

Tobacco was planted in a greenhouse at 25°C, 16 h of light and 8 h of darkness, seeds were sown, and germinated, and when the tobacco grew to the 2-leaf stage, single tobacco was transplanted into a separate flowerpot and was watered every other day to continue cultivation. The experiment can be carried out when the tobacco grows to the 5–6 leaf stage.

### Construction of FTX271 transgenic tobacco

The extraction of total RNA from *F. velutipes* was completed with reference to the instructions of the plant RNA extraction kit (CW Biotech, Nanjing, China). After the concentration of RNA was determined, it was used as a template in the reverse transcription reaction with the HiFiScript cDNA Synthesis Kit (Kangwei Century, Nanjing, Jiangsu, China). The cDNA was then subjected to PCR amplification. The primers for the cDNA sequence of *FTX271* were *FTX271*-F 5’-GCGGATCCACATGCCTCAAGTCAAGACAAG-3′ and *FTX271*-R 5’-GCGGTACCTCACTCAGGACCAGGAACCA-3′, which were designed according to the sequences of TDP (GenBank accession no. AB015948; shown in Supplementary material Table S1). The sequences of GGATCC and GGTACC are the restriction sites of *Kpn I* and *BamH I*.

The purified PCR amplification product was ligated with pUC19-T vector and transformed into DH5α competent cells with reference to the rapid ligation vector kit to construct a cloning vector (Takara, Japan). Single clones were picked and cultured for colony PCR identification. The identified *FTX271* gene was double-digested with *Kpn I* and *BamH I* and inserted into the pROK II plasmid (Miaoling Biotechnology, Wuhan, China) that had been digested with the same restriction enzymes, thus yielding the pROK II-*FTX271* vector.

Using the Agrobacterium-mediated transformation method, the pROK II-*FTX271* vector with correct sequencing was introduced into Agrobacterium GV3101, and the bacterial liquid was identified by PCR and sequencing (Tsingke Biotechnology Co., Ltd., Beijing, China).

Tissue culture was carried out by the tobacco leaf disc method (Pathi et al., 2013), and the operations were performed in order of predifferentiation, coculture, selective culture, rooting culture, seedling cultivation, transplanting and positive strain detection. The seeds of transgenic positive plants (T0 generation) were harvested and continued to be sown, then the target gene of *FTX271* was tested on the T1 and T2 generation plants.

### Detection of the expression of *FTX271* in transgenic tobacco

When the T1 and T2 generation plants grew to the 5–6 leaf stage, total RNA of transgenic tobacco was extracted and subjected to RT–PCR amplification by primers *FTX271*-F and *FTX271*-R. Approximately 50 plants of the T1 and T2 generation were tested.

Total protein of 3 T2 generation tobacco plants that confirmed by RT-PCR were extracted according to the kit instructions (CW Biotech, Nanjing, China). Five milliliters of protein extract (10% protein inhibitor, 90% protein extraction) was added to 1 g of leaves, ground into a homogenate, incubated on ice for 25 min, and then centrifuged at 12000 rpm (4°C, 20 min). The supernatant was concentrated by centrifugation in an ultrafiltration centrifuge tube at 4°C and 12,000 rpm for 20 min. The protein was detected by SDS–PAGE and Western blotting. A polyclonal antibody specific for the C-terminal 20 aa of TDP prepared by GL Biochem (Shanghai, China) and anti-rabbit IgG (HRP conjugated) were used as primary and secondary antibodies, respectively.

### Detection of resistance to TMV in transgenic tobacco

Leaves of T2 generation tobacco plants (*n* = 4) at the 5–6 leaf stage were infected with Agrobacterium suspension containing the TMV-based p35S-30B-GFP vector by a needle-free injector, followed by incubation in the dark for 24 h and then cultured in a greenhouse. To detect TMV multiplication, the expression of GFP was observed and photographed under a 100 W longwave ultraviolet lamp (BlackRaymodel B100ATP, UVP, Upland, CA, USA) on the 1st, 3rd, 5th, 7th, 9th, and 11th days. Wild-type tobacco was treated as a control.

The concentration of TMV CP in each treatment was measured by qRT–PCR, which was performed on the Bio-Rad CFX Manager 3.1 platform, with β-actin of tobacco as an internal reference. Primers were designed according to the sequences of the housekeeping genes β-actin (GenBank: AB158612.1) and TMV CP (GenBank: AY029262.1; Supplementary material Table S1). TMV expression was assessed by evaluating the threshold cycle (CT) values. The relative expression level was calculated using the 2^–ΔΔCT^ method (Li et al., 2019).

### Determination of defense enzyme activities in transgenic tobacco

The experiment was divided into four groups (for three independent biological replicates from different sets of plants; transgenic plant is the T2 generation tobacco): wild-type tobacco (WT), transgenic tobacco (N), wild-type tobacco infected with Agrobacterium suspension containing TMV-based p35S-30B-GFP vector (WT-TMV), and transgenic tobacco infected with Agrobacterium suspension containing TMV-based p35S-30B-GFP vector (N-TMV).

After inoculation with TMV, tobacco leaves were collected on the 1st, 3rd, 5th, 7th, and 9th days, and the total protein was extracted according to the above method. Then, the enzyme activity was detected. Enzyme activity detection was performed according to the kit instructions (Nanjing Jiancheng, Nanjing, China), and phenylalanine ammonia lyase (PAL), polyphenol oxidase (PPO), POD and chitinase (Chitinase) enzyme activities were determined. The OD420 of each sample was measured. Data are expressed as the means ± SDs, *n* = 4 for all groups. To compare differences in means, SPSS statistics 20 was used for analysis of variance (ANOVA). The significance levels for tests were *p* < 0.05.

### Transcriptomic sequencing and bioinformatics analysis

Samples were treated as above, and the upper leaves were collected on the 7th day (four leaves from four different plants per replicate; for three independent biological replicates from different sets of treatments). After treatment with liquid nitrogen, leaves were sent to BGI Tech (Shenzhen, Guangzhou, China) for transcriptome sequencing.

The raw data obtained by sequencing contained reads with low quality (more than 20% of the base qualities were lower than 10), adapter contamination, and a high N content of unknown bases (high N base content more than 5%). Clean reads were obtained by filtering out these reads with Trimmomatic v0.36^1^ software. The filtered data were then mapped to the reference genome of *Nicotiana benthamiana* by HISAT v2.1.0 (Hierarchical Indexing for Spliced Alignment of Transcripts),^2^ followed by new transcript prediction, SNP and InDel and differentially spliced gene detection. New transcripts with protein-coding potential that predicted using CPC v0.9-r2^3^ were added to the reference gene sequence. The gene expression level and transcripts were quantified by RSEM v1.2.8^4^ (Langmead and Salzberg, 2012). The Pearson correlation coefficient between each pair of samples was calculated using the cor function in R software (Supplementary material Figure S1). Read counts obtained from samples were FPKM (fragments per kilobase of exon per million fragments mapped) normalized prior to differentially expressed genes (DEGs) analysis. Based on the gene expression level, differential expression analysis was performed using DEGseq algorithms (parameters: Fold Change ≥1 and Adjusted *p* value ≤0.01), which provided statistical routines for determining digital gene expression data using a model based on the negative binomial distribution.

Finally, the phyper function in R software was used to perform in-depth Gene Ontology (GO) and Kyoto Encyclopedia of Genes and Genomes (KEGG) pathway analyses on the differentially expressed genes, and FDR correction was carried out for the *p* value, with a *Q*-value ≤0.05 as the enrichment standard. Fisher’s exact test was used to screen differentially expressed proteins, and the enrichment criterion was a value of *p* < 0.05. WOLFPSORT was used for the subcellular localization analysis. The GO classification is divided into three functional categories: biological process, cellular component and molecular function.

### Proteome sequencing and bioinformatics analysis

Samples were treated as described above, and the upper leaves were pooled together on the 7th day. Protein extraction and digestion were performed according to Wang et al. (2003), and the peptide was dried under vacuum and stored at −80°C. Mass spectrometry analysis was performed by Hangzhou Jingjie Biological Co., Ltd. (Hangzhou, Zhejiang, China).

Secondary mass spectral data were retrieved using MaxQuant (v1.6.6.0). Search parameter settings: the database is Nicotianna_benthamiana_4100_TX_20190718 (76,475 proteins; Edwards et al., 2017). The reverse library and common contamination library were added to avoid FDR. The peptide digestion method was set to Trypsin/P, and the number of missed cleavage sites was set to 2. The primary precursor ion mass error tolerance of the first search is set to 70 ppm, and the tolerance for the main search is 70 ppm; the mass error tolerance for secondary fragment ions is set to 0.04 Da. Cysteine alkylation was set as a fixed modification, and variable modifications were oxidation of methionine and acetylation and deamidation of the protein N-terminus.

To accurately identify the functions of the differentially expressed proteins, GSEA (Gene Set Enrichment Analysis) was used (Lu et al., 2022). GSEA (4.0.3) is a software developed by the Broad Institute for analyzing pathways enriched by differentially expressed proteins that can scan all pathways, intuitively understanding the difference of all proteins in each pathway, while it does not depend on the *p* value cutoff of differential expression.

To determine the consistency of biological replicates, principal component analysis (PCA) and relative standard deviation (RSD) were used for evaluation, and the results showed that the reproducibility of the samples in this experiment was good (Supplementary material Figure S2). Due to the limited measurement range during protein mass spectrometry analysis, we analyzed the results of peptide lengths in this experiment. The results show that most of the identified peptide lengths are 7–20 amino acids, which conforms to the general rule of trypsin enzymatic hydrolysis and HCD fragmentation (Supplementary material Figure S2).

To better explain the function of differentially expressed proteins (DEPs) in transgenic tobacco and wild-type tobacco, we used InterProScan v.5.14–53.0^5^ software to annotate the screened differential proteins by GO function and used Wolfpsort v.0.2 and CELLO v.2.5 for subcellular localization. To understand the biological functions of differentially expressed proteins, the annotated differential proteins were analyzed by KEGG pathway using KAAS v.2.0^6^ and KEGG v.2.5.^7^

### Validation and function of selected DEGs and DEPs using qRT–PCR

To verify the reliability of the sequencing results, more than 10 pairs of plant resistance-related genes were selected, primers were designed using OLIGO 7.0, and β-actin was used as an internal reference for qRT–PCR validation (Supplementary material Table S1). The detailed process is described above. 2^–ΔΔCT^ was used to calculate the relative expression of the samples during data processing.

## Results

### Construction of transgenic tobacco with *FTX271*

**Figure 1:**
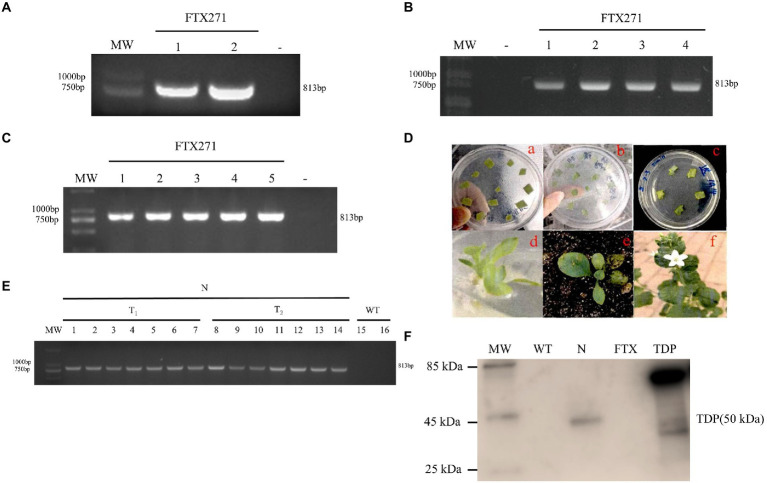
Construction and Validation of *FTX271* transgenic tobacco. **(A)** Agarose gel analysis of the cDNA amplification result of the *FTX271* gene by PCR. **(B)** PCR analysis to determine the insertion of pUC-T-*FTX271* into *E. coli* DH5a. **(C)** PCR analysis of the heat-shock transformation of the pROKII-*FTX271* vector to Agrobacterium GV3101. **(D)** Transformation of tobacco by the Agrobacterium-mediated leaf disc method. The experimental process was as follows: **(a)** predifferentiation; **(b)** coculture; **(c)** selective culture; **(d)** elongation and rooting; **(e)** hardening in a greenhouse; **(f)** transgenic tobacco. For A, B and C, “-” represents the negative water control. **(E)** FTX271-transferred positive verification, WT: wild-type tobacco plants served as the negative control. N: transgenic tobacco. **(F)** Western blot analysis of the expression level of FTX271 in tobacco. Protein extracts from leaves were immunoblotted and probed with antibodies, and protein sizes are shown on the left.

The total RNA extracted from *F. velutipes* was subjected to reverse transcription PCR to obtain a cDNA fragment of approximately 800 bp with the specific primers *FTX271*-F/*FTX271*-R (Figure 1A). Restriction cleavage sites of *BamH I* and *Kpn I* were added to the two terminals of cDNA. After purification, the PCR product was ligated with the pUC19-T vector and transformed into *E. coli* DH5a competent cells. Positive clones were identified by electrophoresis and sequencing (Figure 1B). The results showed that cDNA encodes a protein sequence of 271 aa, which is consistent with the amino acid sequence of Zb (amino acid number AAP38176.1). The aa sequence showed that the anti-TMV protein Zb may be FTX or TDP because its N-end is completely consistent with that of FTX and TDP (Fu et al., 2003).

The sequenced pUC19-T-*FTX271* vector was digested with enzymes of *BamH I* and *Kpn I*, ligated into the plant expression vector pROK II plasmid and transformed into Agrobacterium GV3101 to construct the pROK II-*FTX271* vector. The PCR identification results of the bacterial liquid showed that the vector was successfully transferred into Agrobacterium GV3101 (Figure 1C).

When *N. benthamiana* grows to the 5–6 leaf stage, intact and undamaged young leaves were selected, and the leaf disc method was used for transgenic operations, followed by the steps of predifferentiation, coculture, selective culture, rooting culture, seedling hardening, transplanting, etc. Finally, the transgenic *FTX271* gene in tobacco was successfully constructed (Figure 1D).

### The *FTX271* gene was expressed in transgenic tobacco

The T0 generation seeds of three transgenic tobacco plants were collected and sown. When the T1- and T2-generation plants grew to the 5-6-leaf stage, total RNA of transgenic tobacco was extracted and subjected to RT–PCR amplification by the primers *FTX271*-F and *FTX271*-R (Figure 1E). After sequencing, the aa sequence of the 819 bp PCR product was deduced and was completely consistent with that of TDP. Approximately 50 plants of the T1 and T2 generation were detected, and positive results were obtained in 19.

The total protein extraction of T2 tobacco was completed according to the reagent instructions. A polyclonal antibody specific for the C-terminal 20 aa of TDP and goat anti-rabbit IgG (HRP conjugated) were used as primary and secondary antibodies. Wild-type was used as a negative control, purified FTX and TDP were used as positive controls, and transgenic tobacco protein was analyzed by Western blotting (Figure 1F). Total protein extracted from wild-type tobacco and purified FTX had no bands, while the total protein extracted from transgenic tobacco and purified TDP had, indicating that the *FTX271* gene was exactly transformed and could stably express TDP in *N. benthamiana*, but FTX was not sure.

### Inhibitory activity of the *FTX271* gene against TMV in transgenic plants

**Figure 2:**
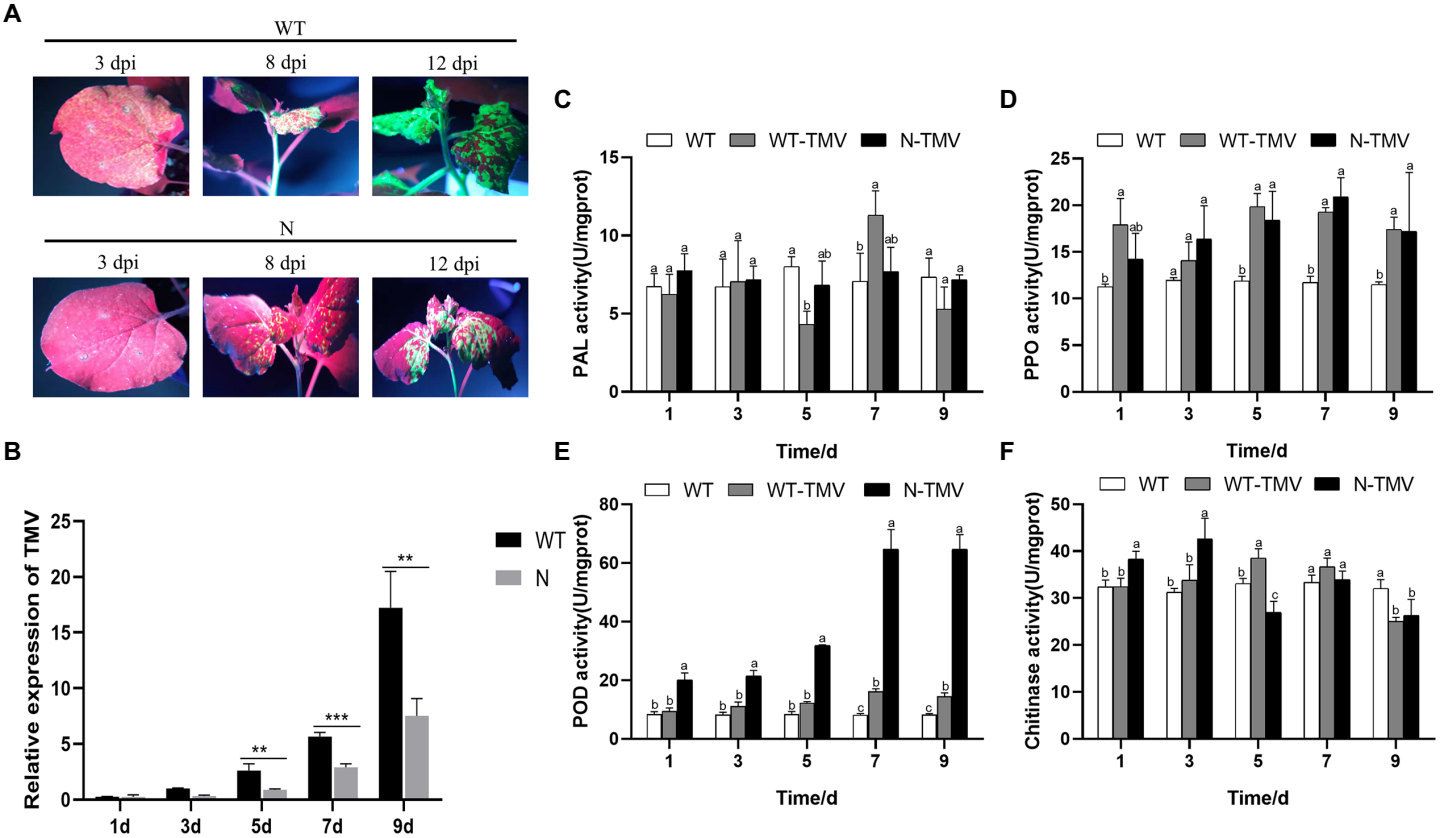
*FTX271* transgenic tobacco exhibited significantly increased resistance to TMV and the activity of defense enzymes. **(A)** Green fluorescent signals representing viral replication in N and WT tobacco plants imaged at the 3rd, 8th, and 12th days postinoculation (dpi). **(B)** Detection of the expression of TMV at 1, 3, 5, 7, and 9 days (dpi) by qRT–PCR. The results are the mean ± SD from three independent experiments. Statistical analysis was performed using Student’s t test (*0.01 < *p* < 0.05, **0.001 < *p* < 0.01, ***p < 0.001). Activity of PAL **(C)**, PPO **(D),** and POD **(E)** and **(F)** chitinase in different treatments on Days 1, 3, 5, 7, and 9 after TMV inoculation (dpi). The experiment was repeated 3 times, and the data presented are the mean ± SD of three independent experiments. Asterisks indicate significant differences between transgenic and WT *N. benthamiana* (Student’s *t*-test, *0.01 < *p* < 0.05, **0.001 < *p* < 0.01, ***p < 0.001). WT, wild type; WT-TMV, wild type inoculated with TMV, N-TMV: transgenic tobacco inoculated with TMV.

The transient expression vector p35S-30B-GFP contains the full-length cDNA sequences of TMV and GFP, which can be expressed soon after transformation into plants. Plasmids of p35S-30B-GFP were transformed into tobacco of wild type (WT) and transgenic lines (N) and then observed under a 100 W longwave ultraviolet lamp. Obvious fluorescence was detected on the 3rd day after leaves of WT were inoculated with p35S-30B-GFP, and it became increasingly stronger over time. At Day 8 after infection, stems also appeared fluorescent, and the fluorescence spread to the whole tobacco plant. The plant exhibited severe shrinkage on the 12th day. However, compared with WT, the fluorescence of transgenic tobacco was much weaker on each day; it was detected on the 5th day, and on the 12th day, fluorescence only appeared on the top leaves and the stem, indicating that the reproduction and transmission of TMV in transgenic tobacco was obviously delayed and suppressed (Figure 2A).

The amount of TMV-CP was further analyzed to investigate the replication status of the virus by qRT–PCR. The results showed that the concentration of TMV-CP in the WT and N groups could be detected on the 3rd and 5th days, respectively, and consistent with the fluorescence strength, the expression level of TMV-CP in the N group was considerably lower than that in the WT at a significance level of 0.01, indicating that *FTX271* could indeed protect tobacco and inhibit the reproduction of TMV (Figure 2B).

### Transfection of *FTX271* increases the activity of defense enzymes in tobacco after TMV infection

To investigate whether defense enzymes are involved in the disease resistance of transgenic tobacco, the activities of four defense-related enzymes, POD, PAL, PPO and chitinase, were detected at different times after p35S-30B-GFP was transformed into tobacco. Although the changing trends were distinguished from each other, some enzyme activities of N-TMV and WT-TMV were always higher than those of the control (WT; Figures 2C–F), indicating that the defense reaction was induced by TMV infection in both transgenic lines and wild type. Although the changing trends of PAL activity, as well as PPO and chitinase, were relatively weak (Figures 2C,D,F), the activity of POD in Groups N-TMV was significantly higher than that in WT-TMV on all days, quickly reached the maximal potency after the 7th day and was nearly 4-fold more potent than the control (Figure 2E). PAL and POD are involved in reactive oxygen species (ROS) catabolism, and they use ROS, including H_2_O_2_ and ‧OH, as substrates in redox reactions and preventing oxidative bursts. The POD activity of transgenic tobacco was greatly increased, which may be due to the increase in ROS content in the body, indicating that this time was in the early stage of the defense response (Yu et al., 2004).

### Overview of transcriptome and proteomics

Symptoms of mosaic appeared in the WT on the 3rd day after inoculation with p35S-30B-GFP, while the symptoms were still not clear in the N lines on the 5th day. Considering the increase in defense-related enzyme activities starting on the 5th day, leaves of the healthy WT, N and TMV treatment samples on the 7th day were selected for transcriptome sequencing and proteomics analysis. Three tobacco plants in each treatment were used as biological repeats.

A total of 567.96 M raw reads were obtained by DNBSeq platform sequencing. After quality control, the clean bases produced an average of 6.39 GB of data, with a Q30 value of more than 92% and a clean read ratio of approximately 90% (Supplementary material Table S2). When the clean reads were compared with the tobacco genome (*N. benthamiana*) using HISAT, the total mapping ratio was 82.33–85.65% (Supplementary material Table S3).

A total of 276,490.0 and 1,256,723.0 secondary spectrograms and 195,372 and 149,357 distinct spectra were obtained by MS in the N, WT, WT-TMV and N-TMV groups. A total of 6750.0 and 6450.0 proteins were identified in this experiment, among which 4752.0 and 4508.0 had quantitative information (Supplementary material Table S4). DEPs were selected with significant changes (*p* value <0.05), and the cutoff point was fixed at >1.5-fold change (*p* value <0.05) for increased abundance proteins and < 0.67-fold change (*p* value <0.05) for reduced abundance proteins. A total of 166 and 26 DEPs were identified, among which 87 and 14 proteins had increased abundance and 79 and 12 proteins had reduced abundance in the N vs. WT and N-TMV vs. WT-TMV, respectively. The target protein FTX271 could be quantified in the transgenic experimental group but not in the WT group.

### Analysis of DEGs and DEPs in N vs. WT lines

Values of log2 FC ≥ 1 and *Q*-value ≤0.01 were used to screen genes with significant differences based on DESEQ2 software. Compared with the healthy WT samples, a total of 953 DEGs were detected in transgenic samples (N), of which 580 were upregulated and 373 were downregulated.

**Figure 3:**
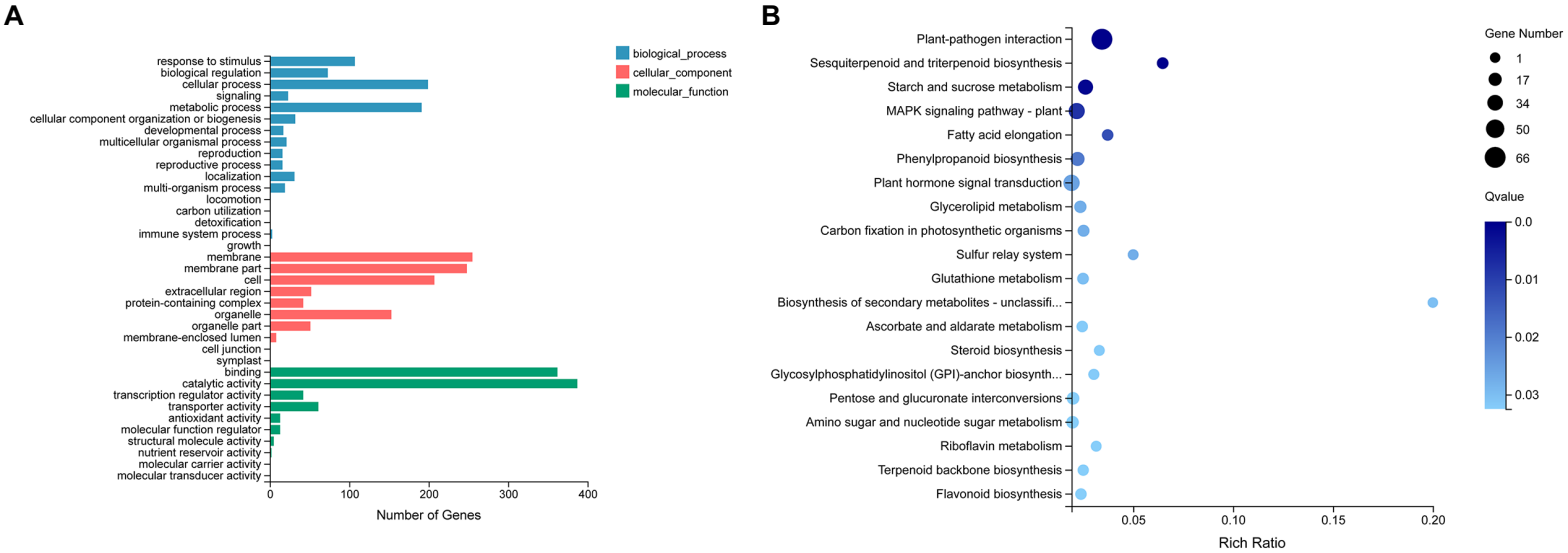
Gene expression analysis of transgenic and WT *N. benthamiana*. **(A)** GO categorization of all differentially expressed genes. The differentially expressed genes were classified into three subontologies: Biological Process, Cellular Component, and Molecular Function. Further KEGG enrichment of the screened differentially expressed genes **(B)**. The size of the bubbles represents the number of genes annotated in a KEGG pathway, and the color represents the enrichment *Q*-value; the darker the color is, the smaller the *Q*-value.

The DEGs in the N vs. WT lines were classified by GO and enriched at different GO functional annotation levels, and the top 20 GO terms are shown in Figure 3A. The results indicated that the DEGs mainly affected metabolism and responses, including glucan endo-1,3-β-D-glucosidase activity, β-glucosidase activity, glucosidase activity, response to stimulus, response to stress, defense response, carbohydrate metabolic process, polysaccharide metabolic process, cellular carbohydrate metabolic process, glucan metabolic process, extracellular region, apoplast, etc. To further clarify the molecular and biological functions of these DEGs in the N vs. WT lines, they were mapped to the KEGG database (*Q*-value ≤0.05; Figure 3B). The results showed that the *FTX271* gene significantly affected pathways related to plant immune function, such as plant pathogen interactions, terpenoid biosynthesis and the MAPK signaling pathway. And the important differentially expressed genes in plant disease resistance-related pathways were analyzed. The expression of genes related to plant disease resistance and involved in plant growth was upregulated, indicating that transfection of the *FTX271* gene can improve the defense response of tobacco and may have an impact on tobacco growth (Supplementary material Table S5).

**Figure 4:**
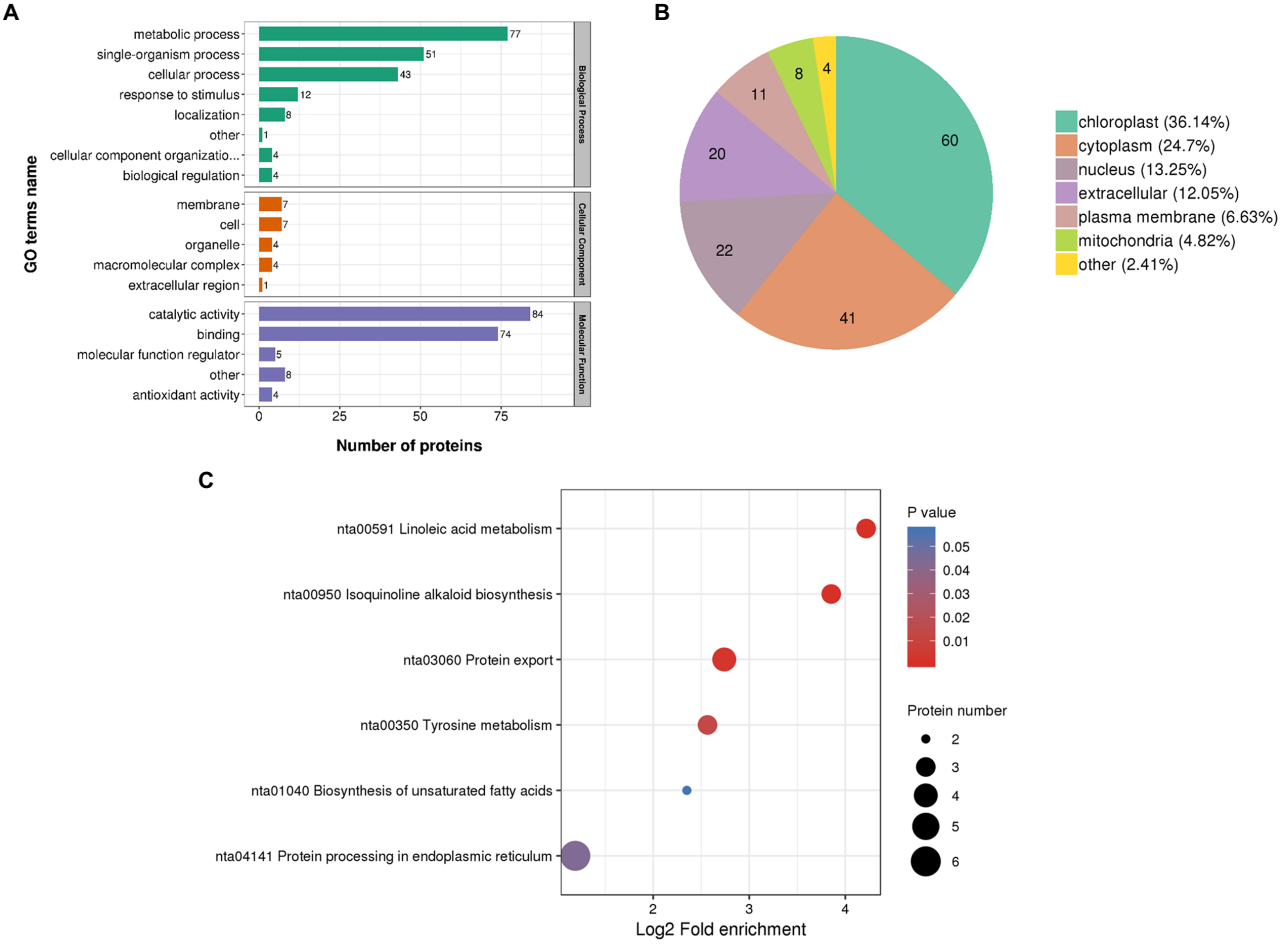
Protein expression analysis of transgenic and WT *N. benthamiana*. **(A)** GO functional annotation was performed on the screened differentially expressed proteins to illustrate the functional status of the differentially expressed proteins of transgenic and WT *N. benthamiana*. **(B)** Subcellular localization of differentially expressed proteins. **(C)** KEGG pathway analysis was performed on the annotated differentially expressed proteins to understand the biological functions of the differentially expressed proteins.

To further clarify the biological functions of 166 DEPs in the N vs. WT groups. GO annotation results indicated that the differential protein expression caused by transfection of *FTX271* is mainly involved in important biological processes, such as plant metabolism and biosynthesis, and has catalytic activity, binding, antioxidant activity and other molecular functions (Figure 4A). The subcellular localization of the differentially expressed proteins was annotated, and the results showed that the differentially expressed proteins were mainly concentrated in the chloroplast, cytoplasm and nucleus, and the upregulated DEPs accounted for a higher proportion in the chloroplast and cytoplasm (Figure 4B). To further understand the biological functions of the DEPs, the annotated differential proteins were subjected to KEGG pathway analysis (Figure 4C), and it was found that the differential proteins were mainly located in the linolenic acid metabolism pathway, isoquinoline alkaloid synthesis, protein export, and tyrosine pathway and in the six signaling pathways of unsaturated fatty acid metabolism and endoplasmic reticulum protein processing. The differential proteins in each signaling pathway were analyzed, and the expression of lipoxygenase (LOX) in the metabolic pathway was upregulated, indicating that *FTX271* can promote the expression of LOX (Supplementary material Table S6). When analyzing the significantly upregulated differential proteins, some proteins related to plant immunity, such as enzymes involved in disease resistance, MAPK proteins involved in autoimmunity, PRs that play an important role in the SAR response, and LOX during JA synthesis, were identified (Supplementary material Table S7).

### Analysis of DEGs and DEPs in N vs. N-TMV and N-TMV vs. WT-TMV lines

**Figure 5:**
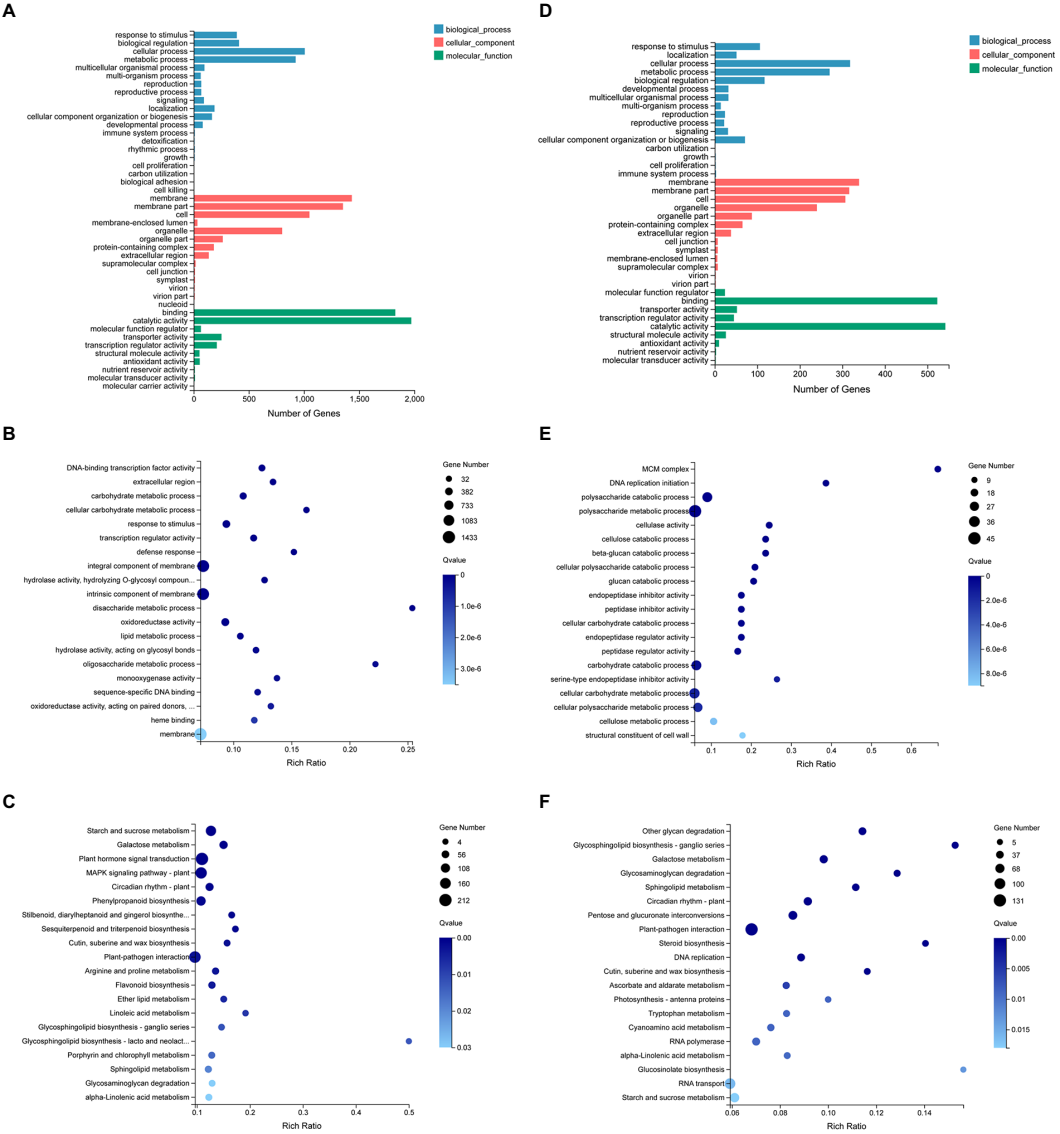
GO analysis and KEGG Pathway of transcriptome differential genes in N vs. N-TMV group and N-TMV vs. WT-TMV group. Go classification of differential genes **(A)**, Bubble plot of GO enrichment of different genes **(B)** and Bubble plot of KEGG Pathway enrichment of different genes **(C)** in N vs. N-TMV group. Go classification of differential genes **(D)**, Bubble plot of GO enrichment of different genes **(E)** and Bubble plot of KEGG Pathway enrichment of different genes **(F)** in N-TMV vs. WT-TMV group.

To explore how *FTX271* transgenic tobacco resist virus invasion, the N vs. N-TMV and WT-TMV vs. N-TMV groups were analyzed by omics. A total of 5,394 differentially expressed genes were screened in the N vs. N-TMV transcriptome, of which 2,997 were upregulated and 2,397 were downregulated. GO classification and enrichment analyses were performed on the DEGs (Figures 5A,B), and it was found that the DEGs were mainly concentrated in the biological pathways of starch and sucrose metabolism and plant hormone signal transduction. The KEGG pathway enrichment results indicated that the transgenic tobacco infected with TMV mainly affected starch and sugar metabolism and phytohormone and glycolipid biosynthesis (Figure 5C).

**Figure 6:**
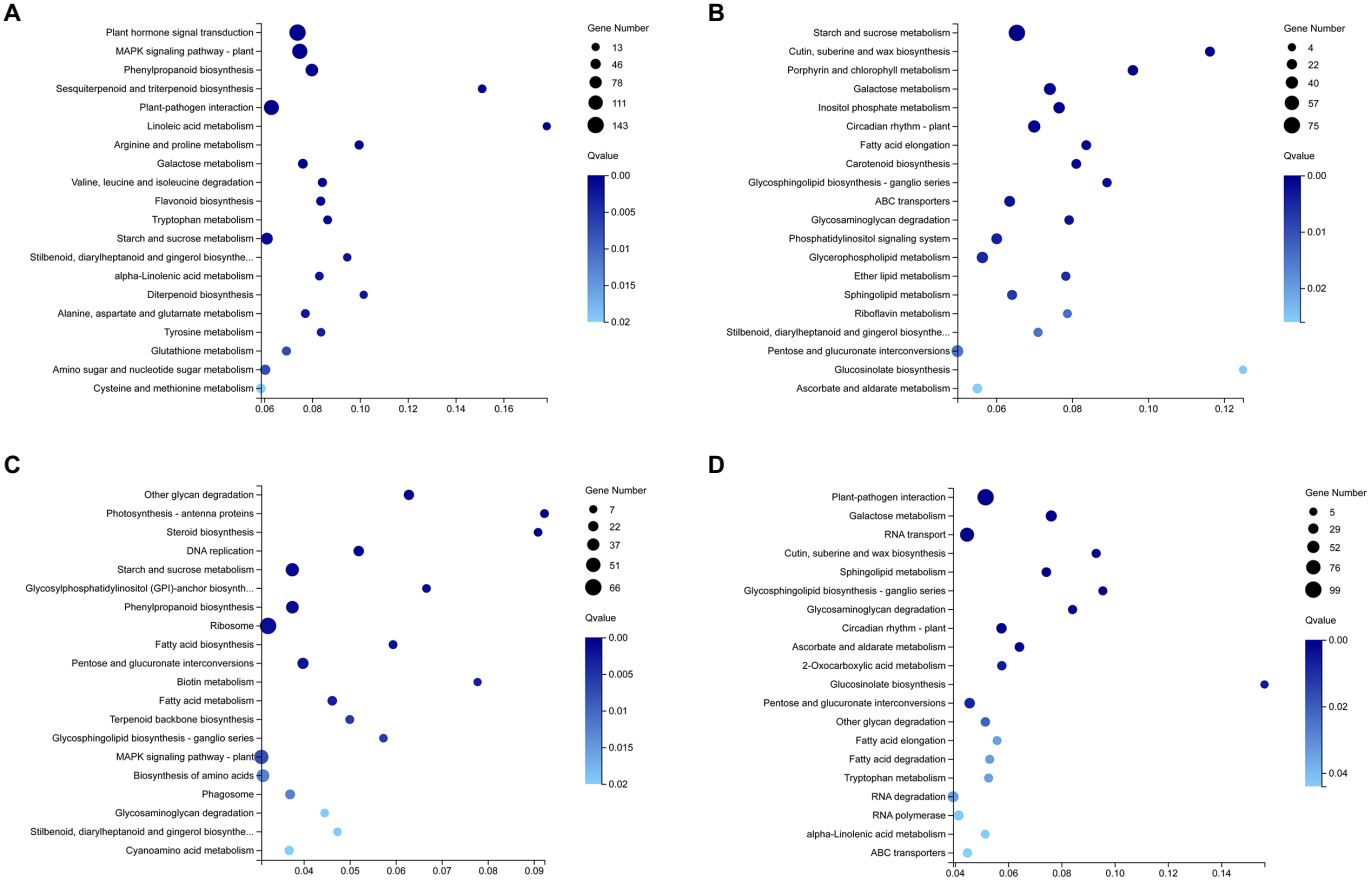
KEGG pathway enrichment of different genes in the N vs. N-TMV group and N-TMV vs. WT-TMV group. KEGG pathway enrichment results of upregulated genes **(A)** and KEGG pathway enrichment results of downregulated genes **(B)** in the N vs. N-TMV group. KEGG pathway enrichment results of upregulated genes **(C)** and KEGG pathway enrichment results of downregulated genes **(D)** in the N-TMV vs. WT-TMV group. For **(A)**–**(D)**, the number of enriched DEGs in the pathway is indicated by the circle area, and the circle color represents the range of the corrected *p*-value.

KEGG pathway enrichment analysis was performed on the upregulated and downregulated genes (Figures 6A,B), and five biological pathways with high enrichment ratios related to plant disease resistance and JA synthesis were found. The related alpha-linolenic acid metabolism and the anabolism of some amino acids were all upregulated, indicating that the upregulated genes mainly affected the defense response of tobacco.

A total of 1,441 DEGs were screened in the transcriptomes of the WT-TMV and N-TMV groups, of which 725 genes were upregulated and 716 were downregulated. GO analysis was performed on the DEGs (Figures 5D,E), and the they were mainly concentrated in the biological pathways of starch and sucrose metabolism and plant hormone signal transduction. The KEGG pathway enrichment results of the DEGs indicated that they mainly affected plant pathogen interactions and glucose metabolism (Figure 5F). The enrichment analysis of the upregulated gene KEGG pathway showed that the photosynthesis-related pathways of transgenic tobacco were upregulated (Figure 6C), suggesting that transfection of *FTX271* may reduce the damage of TMV to chloroplasts by regulating plant hormones to promote photosynthesis or inhibit the accumulation of TMV. The downregulated gene KEGG pathways were enriched in the downregulated plant–pathogen interaction pathway (Figure 6D), indicating that the defense response of transgenic tobacco infected with TMV was milder.

To further clarify the data distribution of the WT-TMV and N-TMV groups, DEPs quantification was represented by a volcano plot (Supplementary material Figure S3A). There were 26 proteins that were significantly differentially expressed, 14 upregulated and 12 downregulated, respectively. Since there were fewer differential proteins quantified by standardized analysis, fewer pathways could be enriched, and the functions of differential proteins could not be accurately identified. Therefore, the GSEA method was used to analyze the differentially expressed proteins. After GSEA, the protein processing pathway in the endoplasmic reticulum (Supplementary material Figure S3B), the upregulated differential proteins were mainly small heat shock proteins (sHSP; Table 1). While sHSP is a defense protein produced by plants under stress, it is speculated that *FTX271* can improve the antiviral ability of transgenic tobacco.

**Table 1:** Partial pathways of GSEA.

Protein accession	Protein description	Sequence coverage (%)	Subcellular localization
**Protein processing in endoplasmic reticulum**			
NbC26232797g0001.1	Class II small heat shock protein Le-HSP17.6II	59.9	Cytoplasm
NbS00004735g0001.1	17.6 kDa classIheat shock protein	45.5	Chloroplast
NbS00037714g0003.1	Heat shock protein 70–3	29.4	Cytoplasm
NbS00002410g0025.1	Mitochondrial small heat shock protein	50.5	Cytoplasm
NbS00001649g0018.1	Class II small heat shock protein Le-HSP17.6	59.1	Cytoplasm
**Photosynthesis**			
NbS00050159g0001.1	Photosystem I reaction center V	17.7	Chloroplast
NbS00033434g0005.1	Oxygen-evolving enhancer protein3-1	18.9	Extracellular
NbS00000833g0023.1	Photosystem I P700 apoprotein A1	14.6	Cytoplasm

### Qrt–PCR validation results are consistent with the omics results

To verify the results of up- and downregulation of important DEGs and DEPs in plant disease resistance-related pathways, 10 differentially expressed genes or proteins were selected from the transcriptome or proteome for qRT–PCR analysis to verify the omics results (Supplementary material Figures S4, S5). For genes with obvious differences or related to disease resistance, some specific gene information is shown in Supplementary material Tables S8, S9, and primers are shown in Supplementary material Table S1. The results showed that the upregulation and downregulation were consistent with the sequencing results.

**Figure 7:**
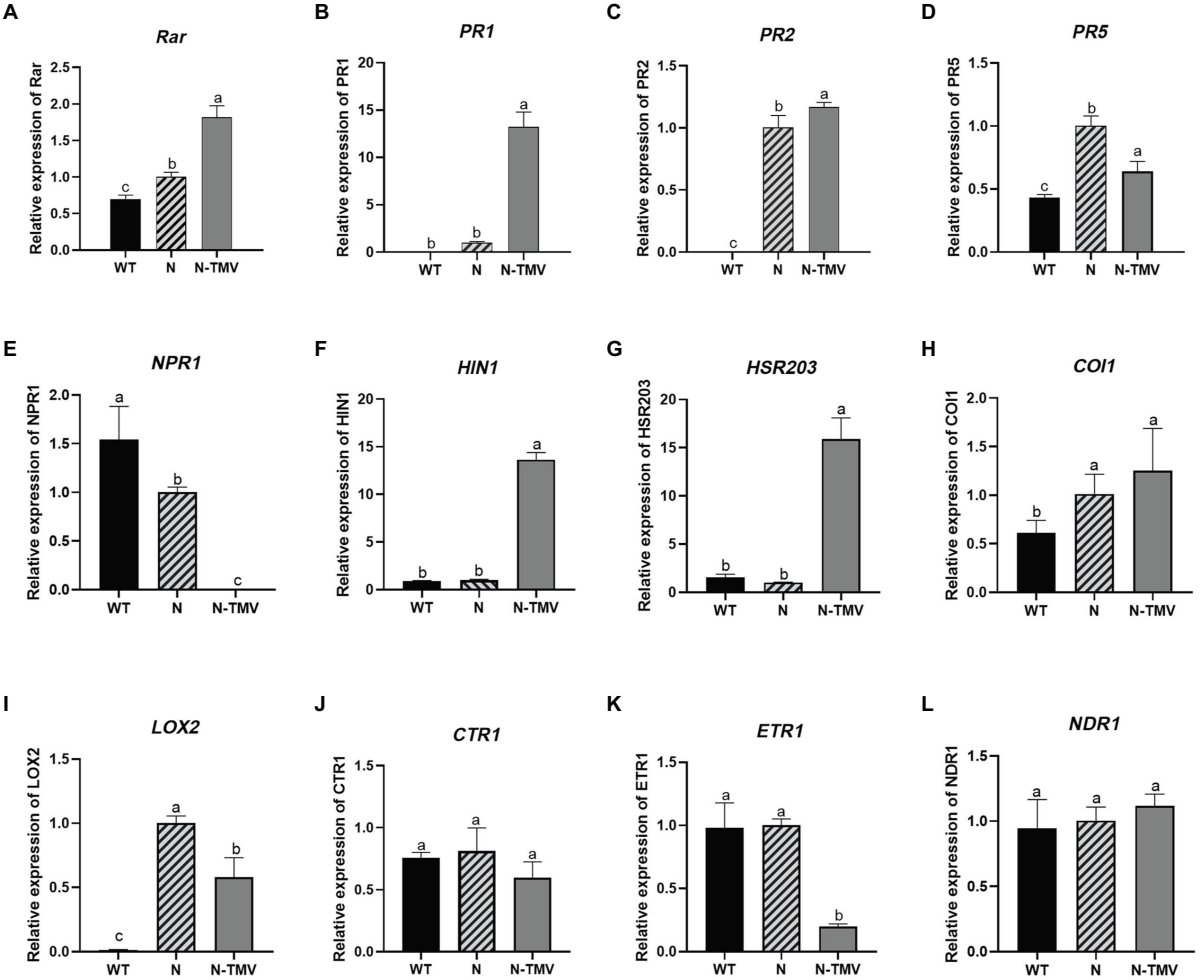
Gene expression analysis of defense genes and the key genes in disease resistance pathways by qRT–PCR. The gene expression levels of *Rar* **(A)**, *PR1* **(B)**, *PR2* **(C)**, *PR5* **(D)**, *COI1* **(E)**, *LOX2* **(F)**, *HIN1* **(G)**, *HSR203* **(H)**, *LOX2* **(I)**, *CTR1* **(J)**, *ETR1* **(K)**, *NDR1* **(N)** in leaves of different treatment groups. Different letters in the same column pattern are significantly different from each other, followed by Duncan’s multiple range test to identify significant differences at *p* < 0.05. Vertical bars refer to the mean ± SD (*n* = 3).

To deeply explore the host defense response of *FTX271* transgenic tobacco to TMV, qRT–PCR was used to detect the expression of a series of defense genes in N, WT, and N-WT (5 dpi). The expression of defense genes showed that the expression levels of *Rar1*, *PR1*, *PR2*, and *PR5* were upregulated in healthy transgenic tobacco compared with wild-type tobacco, indicating that the transfer of *FTX271* promoted the expression of defense genes in *N. benthamiana* and that the expression of defense genes in *N. benthamiana* was increased during infection (Figure 7). The expression levels of *COI1* and *LOX2* in healthy transgenics were higher than those in wild-type tobacco (Figures 7H,I), indicating that *FTX271* activates systemic resistance through the JA pathway. However, the expression levels of the programmed cell death marker genes *H1N1* and *HSR203J* in the two groups of tobacco infected with TMV increased, indicating that TMV infection induced the HR of plants, causing programmed cell death in local areas, and the virus particles were confined to the infection site and activated system resistance.

## Discussion

The systemic immunity of plants, also known as induced resistance, is divided into SAR and ISR (Vlot et al., 2021). Some exogenous substances can stimulate the induced resistance of plants to protect susceptible plants and reduce the damage caused by susceptible factors. For example, both reticine A and PEVD1 can produce induced resistance in tobacco (Wang et al., 2012, 2021).

Our laboratory’s previous *in vitro* experiments have shown that TDP protein from *F. velutipes* can inhibit TMV replication and proliferation and induce SAR by the SA-mediated pathway in common tobacco (Han et al., 2022). In addition, compared with wild-type tobacco, *FTX271* transgenic tobacco not only relieved mosaic symptoms caused by TMV, but also delayed the time of systemic infection (Wu et al., 2017). Here, we would like to explore whether the antiviral mechanism of *FTX271* in transgenic plants is the same as that of TDP *in vitro*.

To explore the effect of the *FTX271* gene on tobacco, we obtained transgenic tobacco (*N. benthamiana*) that can stably express *FTX271* through tobacco leaf disc transformation. TDP is the expression product of the *FTX271* gene in transgenic tobacco. Proteomic differences between transgenic tobacco and wild-type tobacco were analyzed by KEGG annotation analysis. Differential proteins were enriched in metabolic pathways such as linolenic acid metabolism, unsaturated fatty acid metabolism and endoplasmic reticulum protein processing (Figure 4). Differences in each signaling pathway protein analysis showed that the expression of LOX in the metabolic pathway was upregulated, suggesting that *FTX271* could promote the expression of LOX. α-Linolenic acid (α-Lea) is the synthetic precursor of JA, and α-Lea is oxidized by LOX. Upregulation of the linolenic acid metabolism pathway and LOX may lead to an increase in JA content in transgenic tobacco (Ruan et al., 2019). JA, a class of oxygenated lipid derivatives, is a phytohormone necessary for the regulation of plant defense responses (Wasternack and Song, 2017; Ruan et al., 2019). Furthermore, the expression of defense genes measured by qRT–PCR showed that the resistance signal transduction gene *Rar1*, the key gene *COI1* of the JA signaling pathway, and the lipoxygenase gene *LOX2* were upregulated, while the key gene *NPR1* of the salicylic acid-mediated systemic resistance pathway was downregulated. We infer that the transgenic *FTX271* gene induces disease resistance in *N. benthamiana* through the JA pathway to activate ISR in tobacco; thus, the JA signaling pathway plays a major regulatory role in the transgenic tobacco.

Transcriptomic analysis showed that plant hormone signal transduction, the plant Mitogen-activated protein kinase (MAPK) signaling pathway, α-Lea and other metabolic pathways were upregulated after infection with TMV. MAPK cascades play a crucial role in signal transduction in the plant stress response (Lin et al., 2021). Activation of MAPKs also results in multiple defense responses, including defense gene expression and ROS generation (Kroj et al., 2003). The accumulation of ROS acts as a signal to trigger preemptive defense responses, including HR, activating related defense signaling pathways, which can also induce resistance or lead to cell death, depending on the degree of oxidative stress in plants (Apel and Hirt, 2004). HR is a plant defense response triggered by the activation of immune receptors following pathogen recognition (Salguero-Linares and Coll, 2019). Tornero et al. (2002) observed that barley *RAR1* is required for full HR and contributes significantly to HR. *HIN1*, upregulated both during the HR generated by an incompatible plant–pathogen interaction and during senescence, is a marker gene for HR cell death, as well as *HSR203J* (Pontier et al., 1999; Takahashi et al., 2004). The results of qRT–PCR showed that the expression of these genes was upregulated in transgenic tobacco infected with TMV compared with healthy transgenic tobacco (Figure 7), indicating that HR in transgenic tobacco was activated at this time, causing local resistance (Suleman et al., 2021).

**Figure 8:**
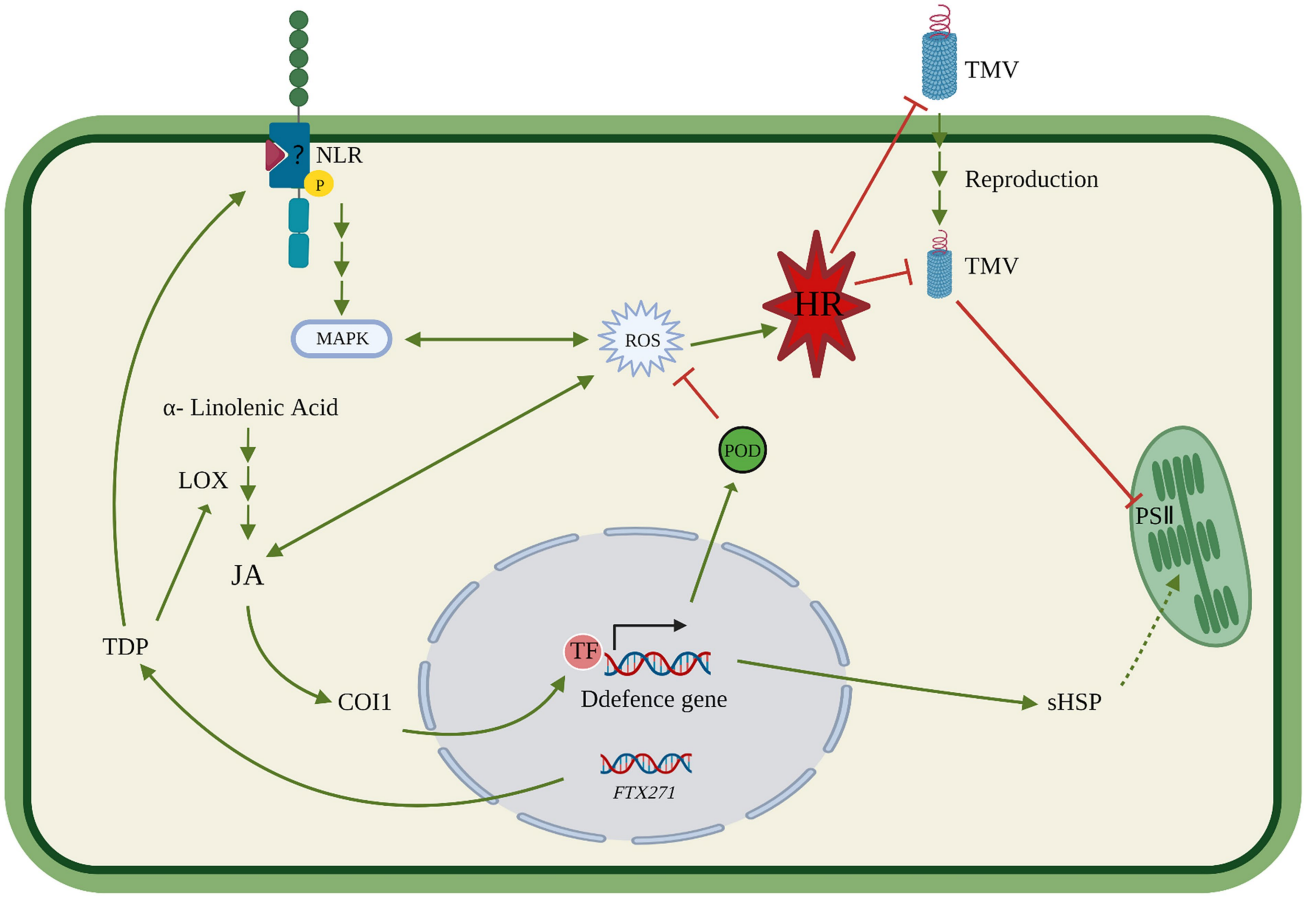
Mechanism of disease resistance in the tobacco trans-*FTX271* gene. 
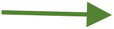
Indicates that the pathway is enhanced. 
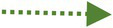
Means an uncertain path. 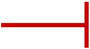
Indicates that the pathway is inhibited. 
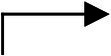
Means gene expression.

A gradual increase in the ROS content would induce an oxidative burst, which may activate the process of senescence. POD is an important oxidoreductase that slows the damage caused by ROS generated during plant HR. The enzymatic activities of the defense enzymes PAL, PPO, POD and chitinase were measured. The results showed that after TMV infection, the POD activity of transgenic tobacco was significantly higher than that of wild-type tobacco, indicating that the plants produced ROS after TMV infection.

Studies have shown that after plants are infected with TMV, the formation of disease is related to the destruction of photosynthesis-related proteins by the virus (Hodgson et al., 1989). In transgenic tobacco, KEGG annotation analysis showed that biological pathways such as photosynthetic antenna proteins, steroid biosynthesis, and terpenoid backbone biosynthesis were upregulated, and photosynthesis-related pathways were also upregulated (Figure 6), which are related to photoprotection (Liu et al., 2020), suggesting that transgenic *FTX271* may promote photosynthesis by regulating plant hormones, thereby delaying and relieving the symptoms of TMV infection of *N. benthamiana*. GSEA of TMV-infected transgenic tobacco and wild-type tobacco and protein processing metabolic pathway analysis in the endoplasmic reticulum found that the upregulated differentially expressed proteins were mainly sHSP. Heat shock proteins act as molecular chaperones that promote the natural folding of proteins and prevent irreversible aggregation of denatured proteins during stress (Li et al., 2015). Meanwhile, in TMV-infected transgenic tobacco, proteomics quantified the upregulated expression of class I sHSP and class II sHSP. Overexpression of CP sHSPs in tobacco can enhance the stability of photosystem II under high-temperature stress (Heckathorn et al., 1998). It is speculated that *FTX271* can induce the expression of sHSP, and transgenic tobacco infected with TMV may enhance the ability of plants to resist TMV by activating the sHSP pathway to compensate for the damage to photosynthesis caused by TMV.

Collectively, TDP expressed in transgenic tobacco acts as an elicitor, activates JA pathway-mediated resistance, enhances the activity of defense enzymes through a signaling cascade, promotes the expression of disease-related proteins, and activates the biosynthesis of hemiterpenoids and triterpenoids so that *FTX271* transgenic tobacco acquires resistance before virus infection. After TMV infects plants, it elicits basic immune responses in transgenic tobacco and activates ROS and HR in tobacco. HR and ROS in transgenic tobacco may cause subsequent systemic resistance responses, and the expression of a series of downstream resistance genes is mediated by JA, thereby inhibiting virus replication and transmission in tobacco. In addition, transgenic tobacco may use sHSP-assisted photoreparation to alleviate the symptoms of TMV (Figure 8). Interestingly, in our previous study, we found that spray TDP on leaves could induced SAR to protect tobacco from TMV infection and alleviate the symptoms caused by TMV. The reasons for the different disease resistance pathways caused by transgenic and spraying need further research and analysis.

In conclusion, JA-mediated ISR and sHSP-assisted photoreparation are activated by *FTX271* to protect tobacco from TMV infection and alleviate the symptoms caused by the virus. The study provided a theoretical basis for the TMV resistance mechanism of *FTX271*, which may represent a potential gene resource for plant antiviral transgenic breeding.

## Data availability statement

The datasets presented in this study can be found in online repositories. The names of the repository/repositories and accession number(s) can be found at: The accession number of the transcriptomic data is SUB11888272 (BioProject database), and the accession number of the proteomics is 1-20220803-135351 (PRIDE database).

## Author contributions

YZ conceptualization, methodology, and writing—original draft. CG data curation and software. YZ visualization and formal analysis. YD and HH investigation and data curation. LaW resources, LiW supervision, resources, writing—review and editing. All authors contributed to the manuscript and approved the submitted version.

## Funding

This study was supported by the National Natural Science Foundation of China (31860525) and Natural Science Foundation of Jiangxi Province (20212BAB205028).

## Conflict of interest

The authors declare that the research was conducted in the absence of any commercial or financial relationships that could be construed as a potential conflict of interest.

## Publisher’s note

All claims expressed in this article are solely those of the authors and do not necessarily represent those of their affiliated organizations, or those of the publisher, the editors and the reviewers. Any product that may be evaluated in this article, or claim that may be made by its manufacturer, is not guaranteed or endorsed by the publisher.

## Supplementary material

The Supplementary material for this article can be found online at: https://www.frontiersin.org/articles/10.3389/fmicb.2022.1003478/full#supplementary-material

## Footnotes

^1^ http://www.usadellab.org/cms/index.php?page=trimmomatic

^2^ http://ftp.solgenomics.net/genomes/Nicotiana_benthamiana/assemblies/

^3^ http://cpc.cbi.pku.edu.cn

^4^ http://deweylab.biostat.wisc.edu/rsem/rsem-calculate-expression.html

^5^ http://www.ebi.ac.uk/interpro/

^6^ http://www.genome.jp/kaas-bin/kaas_main

^7^ http://www.kegg.jp/kegg/mapper.html
