# Supplementary material for: FTX271: A potential gene resource for plant antiviral transgenic breeding
Source: Front Microbiol. 2022 Sep 29;13:1003478. doi: 10.3389/fmicb.2022.1003478 (PMC9558137; doi:10.3389/fmicb.2022.1003478)
Supplement: Supplementary file 1 [file Data_Sheet_1.docx]

Supplementary Material

# Supplementary Figures and Tables

## Supplementary Figures


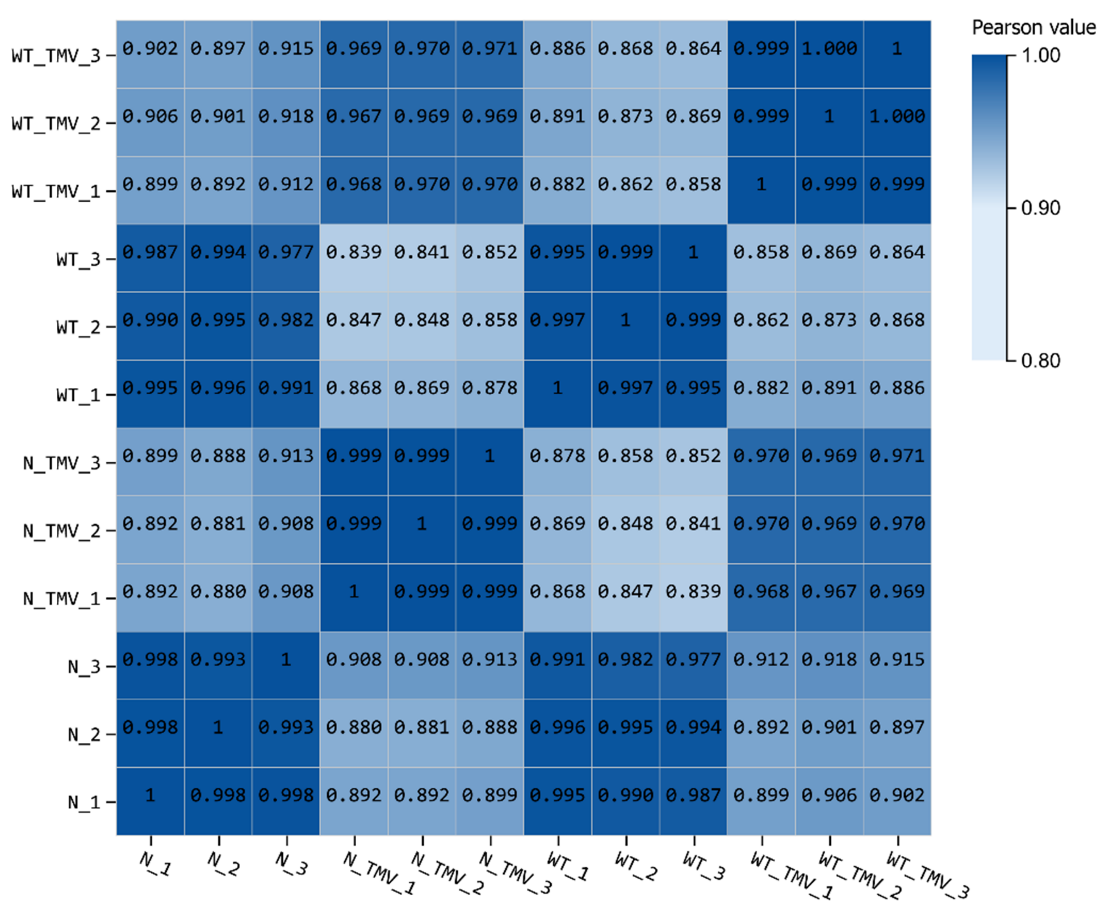


**Figure S1.** Correlation heat map.


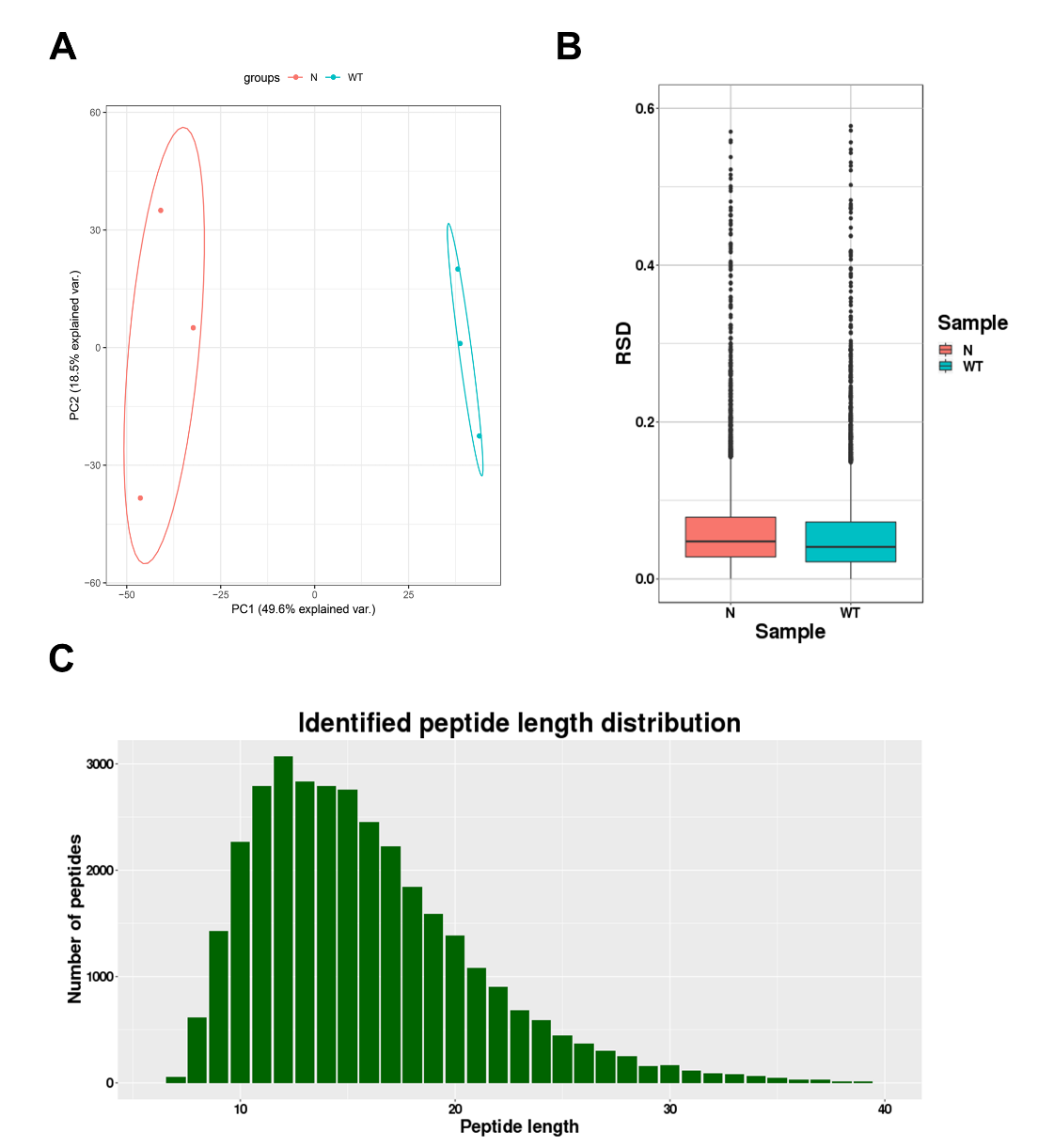


**Figure S2.** Sample repeatability check and length distribution of all identified peptides. **(A)** Principal component analysis test; **(B)** Relative standard deviation test; **(C)** All Identified peptide length distribution.


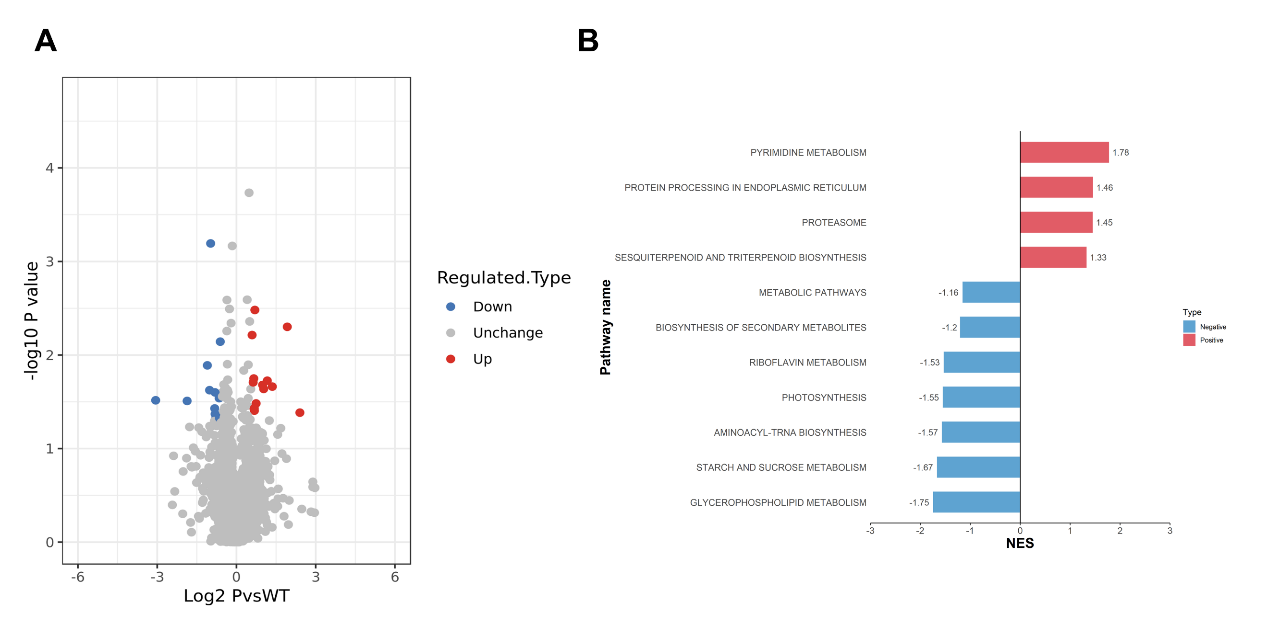


**Figure S3.** Protein expression analysis. The volcano gram of differentially expressed proteins**(A)** and The GSEA analysis of pathways**(B)** in N-TMV-vs-WT-TMV group.


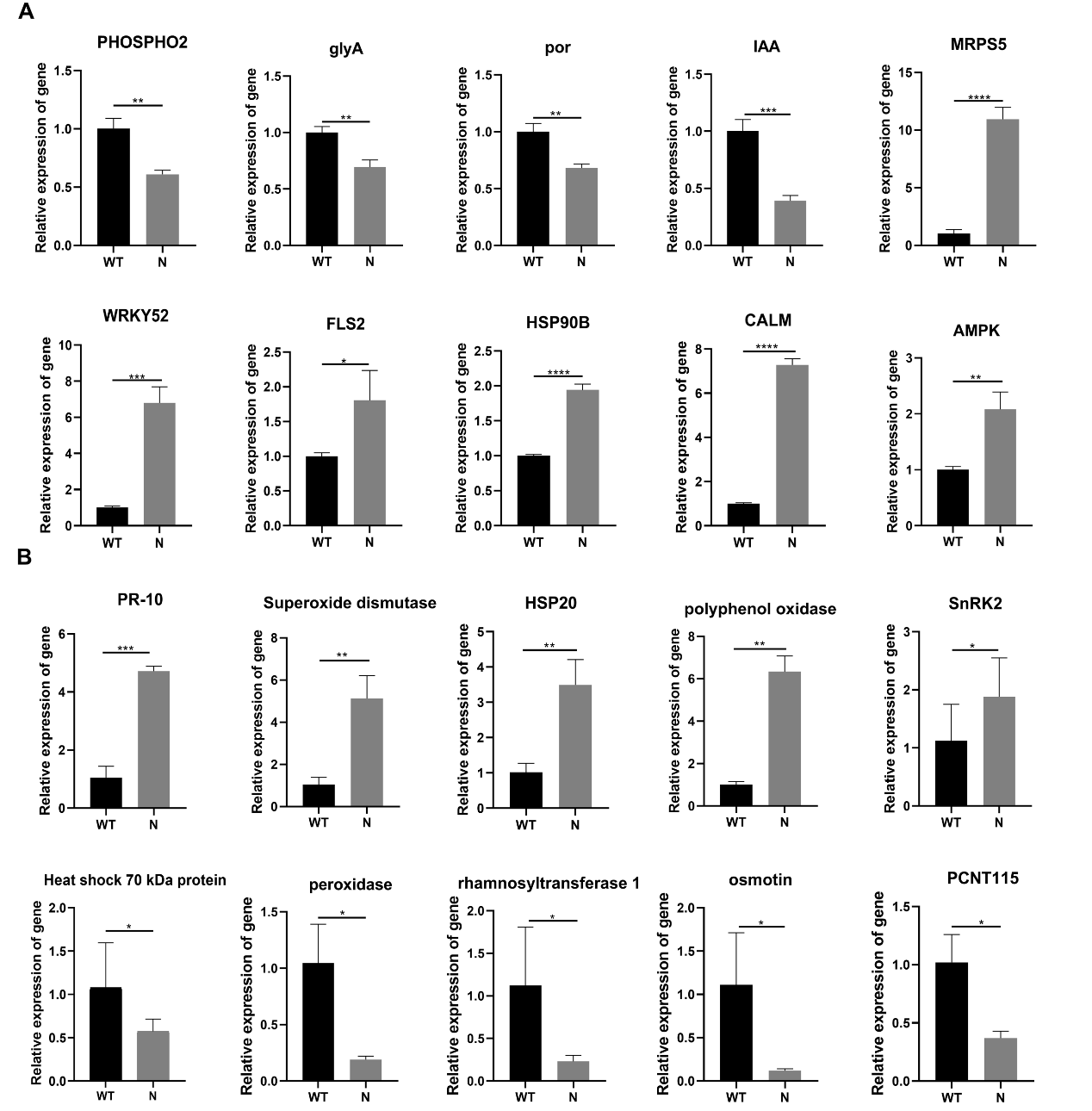


**Figure S4.** Relative expression of genes in WT-vs-N group. Transcriptome-related**(A)** and Proteomee-related**(B)** gene expression. A t test was performed between the control group and other groups: “*”: p value≤0.05, “**”: p value≤0.005, “***”: p value≤0.0005, “NS”: no difference.

**
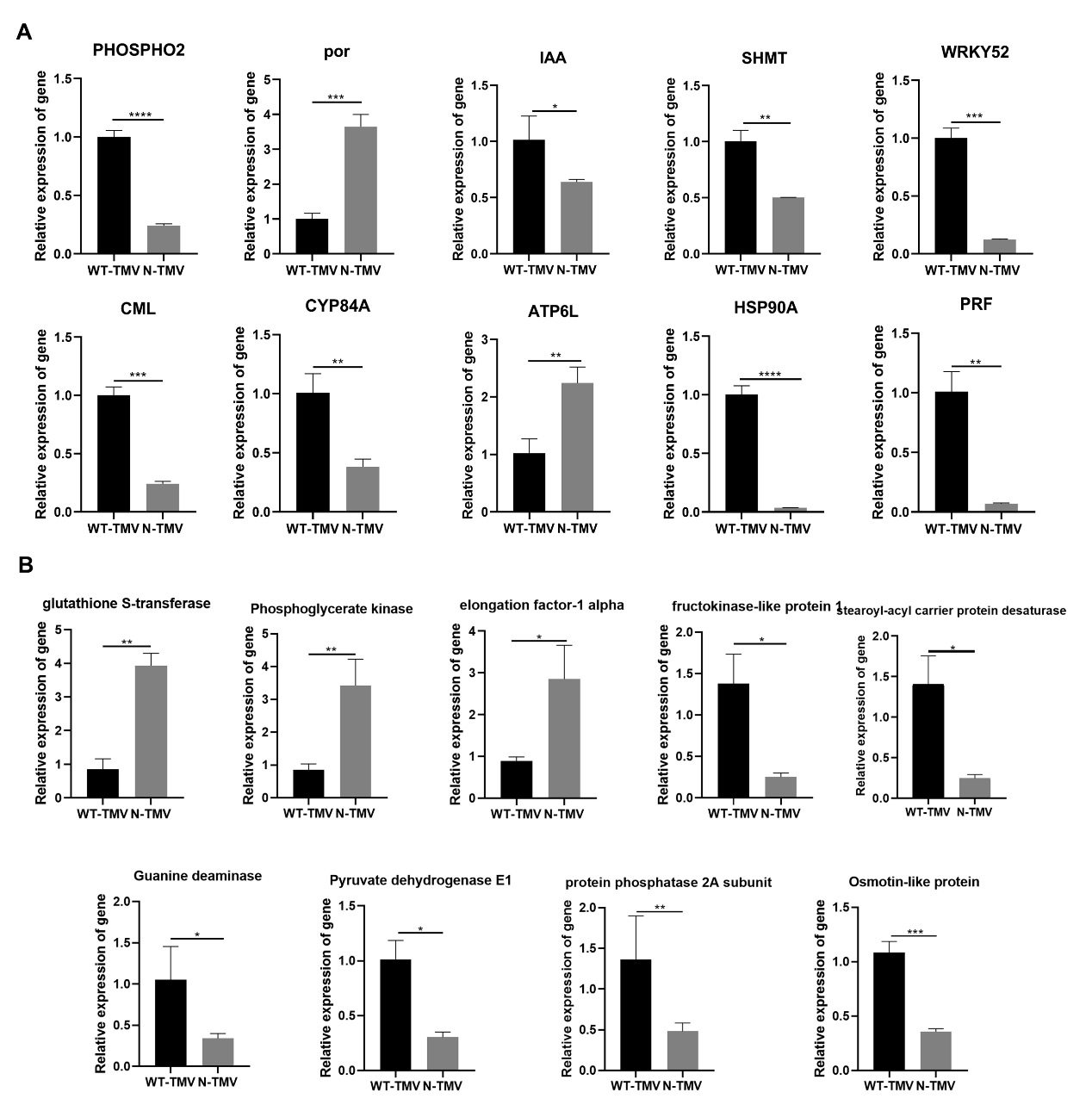
**

**Figure S5.** Relative expression of genes in WT-TMV-vs-N-TMV group. Transcriptome-related**(A)** and Proteomee-related**(B)** gene expression. A t test was performed between the control group and other groups: “*”: p value≤0.05, “**”: p value≤0.005, “***”: p value≤0.0005, “NS”: no difference.

## Supplementary Tables

**Table S1.** List of primers used in this study

| **Genes** | **Primers** | **Sequences (5’ - 3’）** | **Purpose** |
| --- | --- | --- | --- |
| Primers for cDNA sequence of *FTX271* gene | | | |
| *FTX271* | F | ACATGCCTCAAGTCAAGACAAG | PCR |
|  | R | TCACTCAGGACCAGGAACCA |  |
| Primers for partial transcriptome detection genes | | | |
| *Actin* | F | CCTGAGGTCCTTTTCCAACCA | qRT-PCR |
|  | R | GGATTCCGGCAGCTTCCATT |  |
| *PHOSPHO2* | F | AACAATGACAATGGAGGAG | qRT-PCR |
|  | R | ATTAGTAAGGCACAGGGAC |  |
| *por* | F | GCTGCTGCTCTTGTTCCTT | qRT-PCR |
|  | R | TTGTTGCCCTTACTGCTCC |  |
| *IAA* | F | AGACCGTCAAATGCACAAG | qRT-PCR |
|  | R | TTGGCGTCAGATGTCCTCAT |  |
| *WRKY52* | F | TAGGGCTTATTATAGGTGC | qRT-PCR |
|  | R | TTATGGTTTGGTCCAGAGT |  |
| *SHMT* | F | ATTTGCCCAGACTTTAGCG | qRT-PCR |
|  | R | CTTGATGTGAGTGCAGGAG |  |
| *CML* | F | ATCCAGGAAATGATAGAAGC | qRT-PCR |
|  | R | ATTAACTAGCGGAAACCCT |  |
| *ATP6L* | F | AACGAAGCCGAGCACAAAT | qRT-PCR |
|  | R | CCGAGGAAACCGAAGAAGG |  |
| *CYP84A* | F | TTACTATCTCCTAAACCTCCAC | qRT-PCR |
|  | R | TATCATGGGCGTATCTTGT |  |
| *HSP90A* | F | GTATAAAGCTCGTCTTGTCG | qRT-PCR |
|  | R | AACGCTGTCTTAACATCCA |  |
| *PRF* | F | GGCTGAGCATCTTCAATAC | qRT-PCR |
|  | R | ACAATGACAAGAGCCAATC |  |
| Primers for partial proteomic detection genes | | | |
| *PR10* | F | AGTGTGGAGGCCACCTAATTC | qRT-PCR |
|  | R | TTCCAGCTAACAACCGAACCA |  |
| *Superoxide* | F | ATATCCTCCCCAGTTTGCCA | qRT-PCR |
|  | R | TGATAGCCAAAACTCAAGGGATG |  |
| *HSP20* | F | AGAACCAAAGACCACGAGTCC | qRT-PCR |
|  | R | TTGCATCAGTTTCAGGTTTGCT |  |
| *polyphenol* | F | GGGTTGGAAGATGAAGACGC | qRT-PCR |
|  | R | ACAGTAGGCAATCAGACGAAA |  |
| *SnRK2* | F | AGTTGGAAATGAGGCAGTGT | qRT-PCR |
|  | R | AGGTAAACCAAGAGCAACACCA |  |
| *heatS* | F | TTCCCAAGGTTCAACAACTCC | qRT-PCR |
|  | R | CACCAGCAGTTTCAAGACCC |  |
| *peroxidase* | F | TCCTAGCCCCTTTGAAACCC | qRT-PCR |
|  | R | ACGTCGTTCAAAAGTAGCACA |  |
| *UDP* | F | GAAGTAACGGTGAGATGAAGGT | qRT-PCR |
|  | R | CAGTCTAATCCGCCACGTCA |  |
| *osmotin* | F | TTCAATGCTGCAGGTAAAGGT | qRT-PCR |
|  | R | CGTATTCGGCCAAGGTGTTT |  |
| *PCNT115* | F | AGCCTACCTCTTACTGTCTTTCC | qRT-PCR |
|  | R | CTTCTAGCCCCTGTGAACCC |  |
| Primers for various defense genes and TMV | | | |
| *TMV CP* | F | ATCACTACTCCATCTCAGTTC | qRT-PCR |
|  | R | AACAGTTACTTGTGGTGAAGG |  |
| *Rar1* | F | ATGATGGGATGAAGAAGTGG | qRT-PCR |
|  | R | TCGGTGATACATTGGTCGT |  |
| *HIN1* | F | TGCGTCCAGTATTCAAAGGTCA | qRT-PCR |
|  | R | GCTTCACTTCCATCTCATAAACCC |  |
| *HSR203* | F | TGCGTCCAGTATTCAAAGGTCA | qRT-PCR |
|  | R | GCTTCACTTCCATCTCATAAACCC |  |
| *NPR1* | F | TCTATCTTCGATGCGTCTT | qRT-PCR |
|  | R | CTCCTTCAATTCCACCTTA |  |
| *PR1a* | F | GGTGTAGAACCTTTGACCTGGG | qRT-PCR |
|  | R | AAATCGCCACTTCCCTCAGC |  |
| *PR2* | F | TAGAGAATACCTACCCGCCC | qRT-PCR |
|  | R | GAGTGGAAGGTTATGTCGTGC |  |
| *PR4* | F | GTGACGAACACAAGAACAGGAA | qRT-PCR |
|  | R | CCACTCCATTTGTGTCCAAT |  |
| *PR5* | F | CAGAACAAGCTCGATTACG | qRT-PCR |
|  | R | ACATTCACCACCTGCCTAC |  |
| *COI1* | F | AGATCTGCCACTTGATAATG | qRT-PCR |
|  | R | TCTAGAAGGCCTTCATCGG |  |
| *LOX2* | F | GTGCTACAGAGCCATACATC | qRT-PCR |
|  | R | TCAAATCGCCATTCAAGAT |  |
| *CTR1* | F | GCACCACAGGTTGTAGCAG | qRT-PCR |
|  | R | TTAAGATTCGGTCTCAGCA |  |
| *ETR1* | F | ACATCTACCGCCCATACAA | qRT-PCR |
|  | R | CATACCTACCCGTCTCCTC |  |
| *NDR1* | F | AATGCCTTGGAATGATGCT | qRT-PCR |
|  | R | TGCGCTTGATTTACCTGAA |  |

**Table S2.** Data quality statistics of clean reads

| **Sample** | **Total Raw Reads (M)** | **Total Clean Reads (M)** | **Total Clean Bases (Gb)** | **Clean Reads Q20(%)** | **Clean Reads Q30(%)** | **Clean Reads Ratio (%)** |
| --- | --- | --- | --- | --- | --- | --- |
| N_1 | 47.33 | 43.04 | 6.46 | 96.76 | 91.66 | 90.95 |
| N_2 | 47.33 | 42.88 | 6.43 | 97.06 | 92.42 | 90.61 |
| N_3 | 47.33 | 43.04 | 6.46 | 96.63 | 91.34 | 90.94 |
| N_TMV_1 | 47.33 | 43.32 | 6.5 | 96.62 | 91.33 | 91.52 |
| N_TMV_2 | 47.33 | 43.54 | 6.53 | 96.64 | 91.59 | 92.01 |
| N_TMV_3 | 47.33 | 43.21 | 6.48 | 96.83 | 92.04 | 91.3 |
| WT_1 | 47.33 | 42.8 | 6.42 | 97.1 | 92.49 | 90.43 |
| WT_2 | 47.33 | 42.59 | 6.39 | 96.81 | 91.81 | 89.98 |
| WT_3 | 47.33 | 42.49 | 6.37 | 96.92 | 92.09 | 89.78 |
| WT_TMV_1 | 47.33 | 43.42 | 6.51 | 97 | 92.28 | 91.74 |
| WT_TMV_2 | 41.9 | 38.52 | 5.78 | 96.75 | 91.63 | 91.94 |
| WT_TMV_3 | 47.33 | 42.65 | 6.4 | 96.97 | 92.22 | 90.12 |

Sample：sample name; Total Raw Reads (M): The number of reads before filtering; Total Clean Reads (M): Number of filtered reads; Total Clean Bases(Gb): Total number of bases after filtering; Clean Reads Q20(%)：The ratio of the number of bases with a quality value greater than 20 to the total number of bases in the filtered reads (%); Clean Reads Q30(%): The ratio of the number of bases with a quality value greater than 30 in the filtered reads to the total number of bases (%); N: transgenic tobacco group, N-1, N-2, N-3 represent three replicates within the group; N-TMV: TMV-infected tobacco treatment group, N-WTV-1, N-WTV-2, N-WTV-3 represent three replicates within the group; WT: wild-type tobacco group, WT-1, WT-2, WT-3 represent three repeats within the group; WT-TMV: wild-type tobacco treatment group infected with TMV, WT-TMV-1, WT-TMV-2, WT-TMV-3 represent three replicates within the group.

**Table S3.** Statistics of reference genome alignment results

| **Sample** | **Total Clean Reads (M)** | | **Total Mapping (%)** | | **Uniquely Mapping (%)** | |
| --- | --- | --- | --- | --- | --- | --- |
| N_1 | 43.04 | 83.36 | | 15.8 | |  |
| N_2 | 42.88 | 83.71 | | 15.43 | |  |
| N_3 | 43.04 | 83.14 | | 15.87 | |  |
| N_TMV_1 | 43.32 | 84.45 | | 18.7 | |  |
| N_TMV_2 | 43.54 | 84.13 | | 18.94 | |  |
| N_TMV_3 | 43.21 | 83.96 | | 18.72 | |  |
| WT_1 | 42.8 | 85.08 | | 15.4 | |  |
| WT_2 | 42.59 | 85.65 | | 15.21 | |  |
| WT_3 | 42.49 | 85.4 | | 15.21 | |  |
| WT_TMV_1 | 43.42 | 82.47 | | 18.66 | |  |
| WT_TMV_2 | 38.52 | 82.33 | | 18.57 | |  |
| WT_TMV_3 | 42.65 | 82.35 | | 18.54 | |  |

Sample: sample name; Total Clean Reads (M): The total number of Clean reads; Total Mapping (%): the proportion of reference genes in the reads alignment (%); Uniquely Mapping (%): The ratio of the unique position of the reference gene on the reads alignment (%).

**Table S4.** Statistics of reference genome alignment results

| **total number of maps** | **Identification Spectrum** | **Spectral identification rate** | **Number of identified peptides** | **Number of proteins identified** | **Unique Peptides*** |
| --- | --- | --- | --- | --- | --- |
| 276490.0 | 195372 | 70.7% | 33815.0 | 6750.0 | 22329 |

Note: * indicates the number of identified proteins containing at least 2 Unique peptides.

**Table S5.** The differentially expressed genes in in transgenic *FTX271* tobacco and wild-type tobacco

| **Genes accession** | **Genes description** | | **log2Fold**  **Change** | | **Regulated Type** |  |  |
| --- | --- | --- | --- | --- | --- | --- | --- |
| **Plant-pathogen interaction** | | | | | |  |  |
| Niben101Scf01785g10011.1 | LRR receptor-like serine/threonine-protein kinase FLS2 | | 1.286 | | Up |  |  |
| Niben101Scf03114g03011.1 | heat shock protein 82-like | | 3.230 | | Up |  |  |
| Niben101Scf04761g00017.1 | NLR protein required for cell death 2b | | 2.367 | | Up |  |  |
| Niben101Scf07488g00028.1 | disease resistance protein RPS2-like | | 1.263 | | Up |  |  |
| Niben101Scf02195g01001.1 | probable calcium-binding protein CML41 | | 1.495 | | Up |  |  |
| Niben101Scf27914g00006 | Heat shock protein 90 | | 1.078 | | Up |  |  |
| Niben101Scf00107g02001 | toll-interacting protein | | 2.031 | | Up |  |  |
| Niben101Scf00107g03008.1 | pathogenesis-related protein 1 | | 6.412 | | Up |  |  |
| Niben101Scf00539g05012 | calcium-dependent protein kinase | | 1.771 | | Up |  |  |
| Niben101Scf01212g03005.1 | pathogenesis-related genes transcriptional activator PTI5 | | 1.383 | | Up |  |  |
| Niben101Scf01398g00006 | disease resistance protein | | 1.828 | | Up |  |  |
| **MAPK signaling pathway - plant** | | | | | | |  |
| Niben101Scf00107g03008.1 | pathogenesis-related protein 1 | | 6.412 | | Up |  |  |
| Niben101Scf00149g12019 | LRR receptor-like serine/threonine-protein kinase FLS2 | | 2.241 | | Up |  |  |
| Niben101Scf01316g05002 | transcription factor MYC2 | | 2.111 | | Up |  |  |
| Niben101Scf10306g00003.1 | WRKY transcription factor 1 | | 2.184 | | Up |  |  |
| Niben101Scf05057g04016.1 | WRKY transcription factor 2 | | 2.838 | | Up |  |  |
| Niben101Scf01942g04001 | WRKY transcription factor 33 | | 3.537 | | Up | | |
| Niben101Scf03009g03002.1 | WRKY transcription factor 22 | | 1.509 | | Up |  |  |
| Niben101Scf08321g01005 | probable WRKY transcription factor 52 | | 1.118 | | Up |  |  |
| Niben101Scf00367g03013 | ethylene-insensitive protein 3 | | -2.932 | | Down |  |  |
| Niben101Scf04133g02009.1 | calmodulin | | 1.670 | | Up |  |  |
| Niben101Scf07063g00001 | disease resistance protein RPS2 | | 1.392 | | Up |  |  |
| Niben101Scf01742g05007 | interleukin-1 receptor-associated kinase 4 | | -1.134 | | Down |  |  |
| **Plant hormone signal transduction** | | | | | | |  |
| Niben101Scf02530g08015.1 | auxin response factor | | -3.491 | | Down |  |  |
| Niben101Scf03652g03003 | auxin-responsive protein IAA | | -1.862 | | Down |  |  |
| Niben101Scf01316g05002 | transcription factor MYC2 | | 2.111 | | Up |  |  |
| Niben101Scf01742g05007 | interleukin-1 receptor-associated kinase 4 | | -1.134 | | Down |  |  |
| Niben101Scf07563g01004.1 | cyclin D3 | | -1.322 | | Down |  |  |
| Niben101Scf08072g00014 | gibberellin receptor GID1 | | -1.742 | | Down |  |  |
| Niben101Scf08390g07004 | jasmonate ZIM domain-containing protein | | 1.055 | | Up |  |  |
| Niben101Scf13926g00004 | ABA responsive element binding factor | | 1.122 | | Up |  |  |
| **Ascorbate and aldarate metabolism** | | | | | | |  |
| Niben101Scf03026g01009 | L-ascorbate oxidase | | 4.287 | | Up |  |  |
| Niben101Scf03307g06003 | inositol oxygenase | | 2.361 | | Up |  |  |
| **Endocytosis** | | | | | | |  |
| Niben101Scf04490g00001.1 | heat shock 70kDa protein 1/2/6/8 | | 1.429 | | Up |  |  |
| Niben101Scf05412g01004 | clathrin light chain B | | 1.593 | | Up |  |  |
| Niben101Scf08526g01012.1 | Ras-related protein Rab-7A | | -1.178 | | Down |  |  |
| **Ubiquitin mediated proteolysis** | | | | | | |  |
| Niben101Scf02597g01005.1 | E3 ubiquitin-protein ligase PUB23-like | | -1.373 | | Down |  |  |
| **ABC transporters** | | | | | | |  |
| Niben101Scf05166g01006.1 | ABC transporter A family member 2-like | | 1.574 | | Up |  |  |
| Niben101Scf05166g01022 | ABC transporter A family member 7-like | | 1.525 | | Up |  |  |
| Niben101Scf06583g03009 | PDR-type ACB transporter | | 1.533 | | Up |  |  |
| **Antioxidant activity** | | | | | | |  |
| Niben101Scf01702g01012 | peroxidase | | 3.446 | | Up |  |  |
| Niben101Scf02526g00002 | glutathione peroxidase | | -1.111 | | Down |  |  |
| Niben101Scf07070g02002 | alpha-dioxygenase | | 2.952 | | Up |  |  |
| **Detoxification** | | | | | | |  |
| Niben101Scf04174g01004.1 | | glutathione S-transferase | | 2.940 | Up | |  |

**Table S6.** The identified differentially expressed proteins in transgenic *FTX271* tobacco and wild-type tobacco

| **Protein accession** | **Protein description** | **Ratio** | **Regulated Type** | **P value** |
| --- | --- | --- | --- | --- |
| **nta00591 Linoleic acid metabolism** | | | |  |
| NbS00009642g0018.1 | lipoxygenase, partial | 0.189 | Down | 1.768E-07 |
| NbS00000838g0002.1 | lipoxygenase | 2.309 | Up | 1.768E-07 |
| NbS00053961g0017.1 | lipoxygenase | 1.764 | Up | 1.768E-07 |
| **nta00350 Tyrosine metabolism** | | | |  |
| NbS00004173g0007.1 | polyphenoloxidase | 2.589 | Up | 2.585E-07 |
| NbS0000l573g0033.1 | primaryamineoxidase | 0.501 | Down | 0.000215 |
| **nta03060 Protein export** | | | |  |
| NbS00039676g0017.1 | luminalbindingprotein | 0,599 | Down | 8.117E-05 |
| NbS00040865g0006.1 | Luminal-bindingprotein | 50.605 | Down | 1.38E-06 |
| NbS00043310g0001.1 | innermembraneprotein | 0.648 | Down | 0.0068439 |
| NbS00Q31872g0001.1 | luminalbindingprotein | 0.573 | Down | 0.0003173 |
| **nta00950 Isoquinoline alkaloid biosynthesis** | | | |  |
| NbS00004173g0007.1 | polyphenoloxidase | 2.589 | Up | 2.585E-07 |
| NbS00001573g0033.1 | primaryamineoxidase | 0.501 | Down | 0.000215 |
| **nta04141 Protein processing in endoplasmic reticulum** | | | |  |
| NbS00039676g0017.1 | luminalbindingprotein | 0.599 | Down | 8.117E-05 |
| NbS00014052g0002.1 | proteindisulfideisomerase | 0.453 | Down | 1.772E-05 |
| NbS00040865g0006.1 | Luminal-bindingprotein | 50.605 | Down | 1.38E-06 |
| NbS0QQ31872g0001.1 | luminalbindingprotein | 0.573 | Down | 0.0003173 |
| **nta01040 Biosynthesis of unsaturated fatty acids** | | | |  |
| NbS00017709g0003.1 | stearoyl-acylcarrierprotein desaturase | 0.554 | Down | 0.0001812 |
| NbS00Q06179g0007.1 | 3-ketoacylCoAthiolase1 | 1.546 | Up | 0.0001199 |

**Table S7.** The identified up-regulated differentially expressed proteins in transgenic *FTX271* tobacco and wild-type tobacco (Ratio>1.5, p<0.05)

| **Protein accession** | **Protein description** | **Ratio** | **P value** | Sequence coverage  (%) |
| --- | --- | --- | --- | --- |
| NbS00034633g0005.1 | Superoxide dismutase PR-10 type | 3.181 | 0.0003048 | 60.9 |
| NbS00038554g0002.1 | pathogenesis-related protein | 4.489 | 0.00048408 | 56.8 |
| NbS00029284g0008.1 | threoninedeaminase | 2.739 | 1.537E-05 | 50.2 |
| NbS00004173g0007.1 | polyphenoloxidase | 2.589 | 2.585E-07 | 36.8 |
| NbS00010129g0001.1 | Glucan endo-l，3-beta-glucosidase | 4.181 | 2.323E-06 | 35.5 |
| NbS00053961g0017.1 | Lipoxygenase | 1.764 | 2.488E-05 | 23.1 |
| NbS00038096g0016.1 | mitogen-activated protein kinase 6 | 1.84 | 5.921E-05 | 17.5 |
| NbS00008330g0013.1 | trehalose synthase | 1.513 | 0.0161564 | 17.2 |
| NbS00013780g0007.1 | peroxidase | 1.783 | 0.0006794 | 15.6 |

Note: Ratio: the relative quantification value of protein in transgenic tobacco and wild-type tobacco is the difference fold; Sequencecoverage: The percentage of identified peptides to the total peptides.

**Table S8.** Gene information of some genes in transcriptome

| **Gene accession** | **Gene description** | **log2FoldChange** |
| --- | --- | --- |
| The gene information of the transcriptomics uninfected TMV | | |
| Niben101Scf09321g01008.1 | PHOSPHO2 | -0.9 |
| Niben101Scf01171g03015.1 | glyA | -0.27 |
| Niben101Scf00506g02003 | por | -0.26 |
| Niben101Scf11535g01026 | IAA | -0.53 |
| BGI_novel_G000712 | MRPS5 | 0.68 |
| Niben101Scf01076g02011 | WRKY52 | 0.8 |
| Niben101Scf01785g10011.1 | FLS2 | 1.29 |
| Niben101Scf27914g00006 | HSP90B | 1.08 |
| Niben101Scf04133g02009.1 | CALM | 1.67 |
| BGI_novel_G000586 | AMPK | 1.99 |
| The gene information of the transcriptomics infected TMV | | |
| Niben101Scf09321g01008.1 | PHOSPHO2 | -1.74 |
| Niben101Scf00506g02003 | por | 1.17 |
| Niben101Scf11535g01026 | IAA | -0.33 |
| Niben101Scf07073g02007.1 | SHMT | -0.37 |
| Niben101Scf01076g02011 | WRKY52 | -1.23 |
| Niben101Scf00751g01008 | CML | -0.56 |
| Niben101Scf01433g07012.1 | CYP84A | -0.22 |
| Niben101Scf03735g07010.1 | ATP6L | 1.67 |
| BGI_novel_G000413 | HSP90A | -2.2 |
| BGI_novel_G000809 | PRF | -1.58 |

**Table S9** The gene information of the proteomics partial genes

| **Protein accession** | **Protein description** | **Ratio** |
| --- | --- | --- |
| NbS00038554g0002.1 | putative PR-10 type pathogenesis-related protein | 4.489 |
| NbS00034633g0005.1 | Superoxide dismutase [Cu-Zn] | 3.183 |
| NbS00007106g0007.1 | HSP20-like chaperones superfamily protein | 3.089 |
| NbS00004173g0007.1 | polyphenol oxidase | 2.589 |
| NbS00043104g0004.1 | SnRK2 calcium sensor | 1.729 |
| NbS00039744g0003.1 | Heat shock cognate 70 kDa protein 2 | 0.633 |
| NbS00019371g0003.1 | peroxidase | 0.527 |
| NbS00025506g0001.1 | putative UDP-rhamnose: rhamnosyltransferase 1 | 0.335 |
| NbS00026613g0001.1 | osmotin | 0.161 |
| NbS00009622g0029.1 | Auxin-induced protein PCNT115 | 0.523 |
